# Exceptional avian herbivores: multiple transitions toward herbivory in the bird order Anseriformes and its correlation with body mass

**DOI:** 10.1002/ece3.1787

**Published:** 2015-10-15

**Authors:** Aaron M. Olsen

**Affiliations:** ^1^ Department of Organismal Biology and AnatomyUniversity of Chicago1027 E. 57th StreetChicagoIllinois60637; ^2^ Bird DivisionThe Field Museum of Natural History1400 S. Lake Shore DriveChicagoIllinois60605

**Keywords:** Aves, behavior, diet evolution, life‐history evolution, macroevolution, morphological evolution

## Abstract

Herbivory is rare among birds and is usually thought to have evolved predominately among large, flightless birds due to energetic constraints or an association with increased body mass. Nearly all members of the bird order Anseriformes, which includes ducks, geese, and swans, are flighted and many are predominately herbivorous. However, it is unknown whether herbivory represents a derived state for the order and how many times a predominately herbivorous diet may have evolved. Compiling data from over 200 published diet studies to create a continuous character for herbivory, models of trait evolution support at least five independent transitions toward a predominately herbivorous diet in Anseriformes. Although a nonphylogenetic correlation test recovers a significant positive correlation between herbivory and body mass, this correlation is not significant when accounting for phylogeny. These results indicate a lack of support for the hypothesis that a larger body mass confers an advantage in the digestion of low‐quality diets but does not exclude the possibility that shifts to a more abundant food source have driven shifts toward herbivory in other bird lineages. The exceptional number of transitions toward a more herbivorous diet in Anseriformes and lack of correlation with body mass prompts a reinterpretation of the relatively infrequent origination of herbivory among flighted birds.

## Introduction

Herbivory is exceptional among dietary strategies in that it exploits one of earth's most abundant, and yet least digestible, food sources: the leaves, stems, buds, and bulbs of plants, (Karasov 1990). Despite the associated digestive challenges, herbivory has evolved repeatedly in several vertebrates, including mammals (Cork 1996; Janis 2000), turtles (Bjorndal 1980), lizards (Cooper and Vitt 2002; Stayton 2006), and teleost fishes (Roberts 1987; Winterbottom and McLennan 1993; Choat and Clements 1998). Although only 2% of extant bird species have a diet that is primarily herbivorous, herbivory has likely evolved in birds at least nine times (Table 1). These independent origins appear to show little phylogenetic pattern. Origins of herbivory are scattered across the avian tree of life and it is commonly assumed that herbivory has evolved no more than twice within any one order. In contrast to this lack of phylogenetic pattern, there does appear to be an association between herbivory and lower flight capacity or the loss of flight entirely. Most herbivorous ratites, the Takahe (*Porphyrio mantelli*) and the Kakapo (*Strigops habroptila*), are flightless. Although Galliformes, Tinamiformes and the Hoatzin (*Opisthocomus hoazin*) are capable of flight, flight in these groups is characterized by short, explosive bouts used primarily in escape from predators (Dial 2003) or short‐range travel (up to 350 m in the Hoatzin; Strahl 1988). Among avian herbivores, herbivorous Anseriformes are exceptional in being generally strong fliers with the capacity for long‐range flight, albeit poor maneuverability and a narrow range of flight speeds (Dial 2003).

**Table 1:** Origins of herbivory in birds

Order	Num origins	Total Spp	Flight?	Examples	Reference
Ratites	1–2				
Struthioniformes^a^		<5^b^	No	Ostrich (*Struthio camelus*)	Milton et al. (1994)
				Emu (*Dromaius novaehollandiae*)	Davies (1978)
+ Tinamiformes		<47	Escape^c^	Andean Tinamou (*Nothoprocta pentlandii*)	Mosa (1993)
Dinornithiformes (Moas)^d^		9	No		Wood et al. (2008)
Aepyornithiformes (Elephant birds)^d^		2–7	No	Elephant bird (*Aepyornis*)	Clarke et al. (2006)
Galliformes	1+	<19^e^	Escape^c^	Rock Ptarmigan (*Lagopus muta*)	Sedinger (1997)
Anseriformes	1+	20–30	Yes	Screamers, geese, swans, moa‐nalos	Morton (1978) James and Burney (1997)
Opisthocomiformes	1	1	Weak^f^	Hoatzin (*Opisthocomus hoazin*)	Grajal et al. (1989)
Gruiformes^a^	1	1	No	Takahe (*Porphyrio mantelli*)	Mills and Mark (1977)
Psittaciformes	1	1	No	Kakapo (*Strigops habroptila*)	Trewick (1996)
Passeriformes	2+	2	Yes	White‐tipped Plantcutter (*Phytotoma rutila*)	Bucher et al. (2003)
				Thick‐billed Saltators (*Saltator maxillosus*)	Munson and Robinson (1992)
Totals	9+	<115			

Examples include species for which leaves, stems, buds, and bulbs comprise greater than 80% of stomach contents or foraging observations for at least a season.

a Likely nonmonophyletic (Hackett et al. 2008; Mitchell et al. 2014).

b Includes all those in *Struthio*,* Rhea*, and *Dromaius*.

c Fly in brief, explosive escape bouts (Dial 2003).

d Extinct.

e Includes all species in Tetraonidae.

f Observed to fly up to 350 m without rest (Strahl 1988).

It is often assumed that herbivory is observed primarily among flightless and weak‐flying birds due to a potential association with an increase in body mass (Morton 1978; Dudley and Vermeij 1992; Klasing 1998), which places an increased cost on flight (Masman and Klaassen 1987; e.g. Guillemette 1994). Two main hypotheses propose mechanisms by which herbivory may be positively associated with body mass. The first, based initially on the Jarman–Bell Principle (Geist 1974), posits a physiological mechanism whereby parameters that enhance digestive capacity, such food intake rate, scale with body mass at a rate greater than energy expenditure (basal metabolic rate), thus affording larger individuals an energetic advantage when feeding on low‐quality foods relative to smaller individuals. This hypothesis, referred to here as the “digestive efficiency” hypothesis, predicts that within already herbivorous lineages, larger individuals will outcompete smaller individuals or reach a degree of herbivory not possible at lower body masses, leading to an observed positive correlation between herbivory and body mass. A second hypothesis proposes an ecological mechanism whereby body size is not positively associated with herbivory, per se, but rather with diets that provide sufficiently abundant and large packets of food (Hiiemae 2000; Clauss et al. 2013). This hypothesis, referred to here as the “abundance‐packet size” hypothesis, predicts that as a lineage increases in body mass, selection will act to shift the diet toward foods that are available in larger or more abundant packets, such as leaves of plants, resulting in an observed positive correlation between herbivory and body mass.

Herbivorous birds tend to have larger body masses than their closest relatives: the Takahe is among the 15 heaviest birds in the order Gruiformes, the Kakapo and swans are the heaviest members of their orders, and Ostriches and Emus are the heaviest and sixth heaviest extant birds, respectively (Dunning 2008). Studies in lizards (Pough 1973; Schluter 1984; Cooper and Vitt 2002) and terrestrial mammals (Price and Hopkins 2015) have found positive correlations between body mass and herbivory. However, studies in herbivorous primates (Clauss et al. 2008), reptiles (Franz et al. 2011), ungulates (Steuer et al. 2011), and herbivorous mammals more generally (Clauss et al. 2007; Müller et al. 2013) have found little or no empirical support for the digestive efficiency hypothesis, indicating that such correlations are not due to any digestive advantage conferred by an increase in body mass.

For birds in particular, known allometries of digestive parameters do not support the digestive efficiency hypothesis. The scaling of food intake rate to body mass does not differ significantly from that of basal metabolic rate to body mass (Fritz et al. 2012) and the length of time food is retained in the gut, which is positively associated with the extent of digestion (Demment and Van Soest 1985; Prop and Vulink 1992), is uncorrelated with body mass among herbivorous birds (Fritz et al. 2012). Although larger birds have larger small intestinal volumes relative to their basal metabolic rate (Lavin et al. 2008), it is unlikely that such scaling would contribute an increase in digestive efficiency for herbivores given that large intestine and cecal volumes (for which few morphological data are available) bear greater relevance to the digestion of high fiber foods (Leopold 1953; McBee and West 1969; Gasaway 1976; Barnes and Thomas 1987). Thus, any potential correlation between herbivory and body mass in birds is more likely due to an ecological, rather than physiological, mechanism.

**Figure 1:**
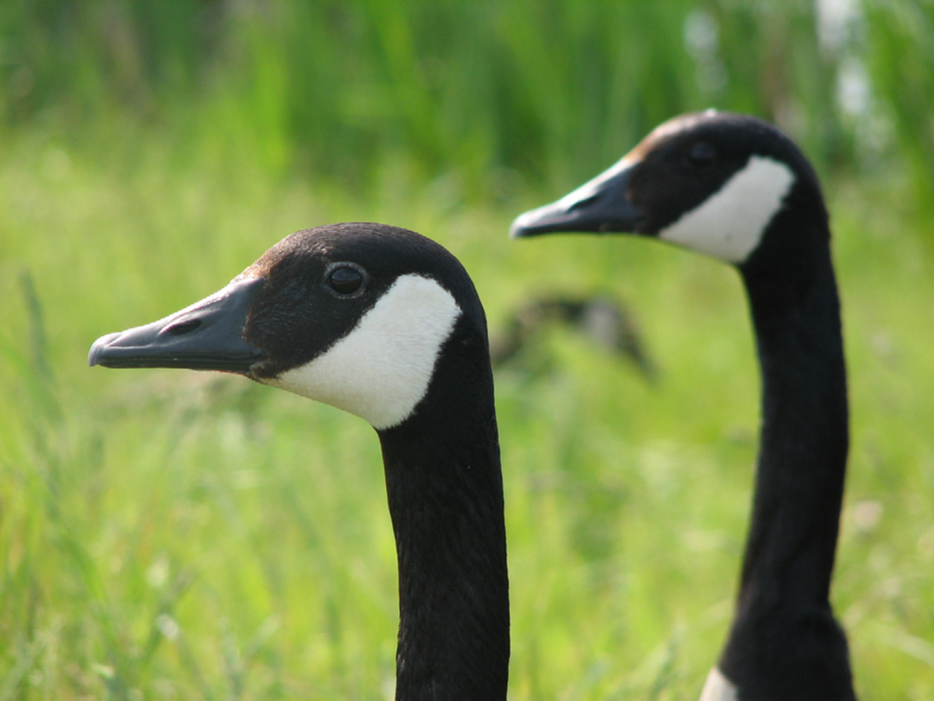
Canada Geese (*Branta canadensis*) feeding on grasses in Sarpy County, Nebraska. Canada Geese have partially herbivorous diets, composed primarily of the leaves, seeds, and fruits of plants.

Birds in the order Anseriformes, which includes ducks, geese, swans, and mergansers, consume a diversity of foods: seeds, fish, mollusks, aquatic insects, algae, and herbage of aquatic and terrestrial plants (Fig. 1). While herbivory is most prevalent among those Anseriformes commonly known as geese (Lavery 1971; Middleton and van der Valk 1987; Kingsford 1989), it is also observed in screamers (Naranjo 1986), the sister group to all other Anseriformes, swans (Squires and Anderson 1995; Bailey et al. 2008), and some species of ducks, such as the American Wigeon (*Anas americana*; Wishart 1983; Havera 1998). Additionally, nearly all Anseriformes are flighted, with the exception of three species of steamer ducks and the extinct moa‐nalos (Olson and James 1991; Fulton et al. 2012). It has long been recognized that ducks and geese are both polyphyletic (Delacour and Mayr 1945) and this has been supported by phylogenetic studies based on morphological (Livezey 1986) and molecular (Sorenson et al. 1999; Donne‐Goussé et al. 2002; González et al. 2009) characters. The polyphyly of geese and the occurrence of herbivory in several other anseriform taxa raise the question of how many times herbivory has evolved in Anseriformes. If an herbivorous diet has evolved repeatedly, this would provide an ideal system in which to test whether increased herbivory is associated with an increase in body mass in birds.

In this study, I examine the evolution of herbivory in Anseriformes, using quantitative and qualitative diet data compiled from the literature to estimate the degree of herbivory for most anseriform species. I reconstruct the ancestral degree of herbivory at key nodes in the anseriform phylogeny using models of continuous trait evolution. I then test whether herbivory is significantly correlated with body mass using nonphylogenetic and phylogenetic correlation tests. I explore the effects of different phylogenetic relationships, branch lengths, and definitions of herbivory. Lastly, I discuss the implications of this study's results for the origins of geese, the relationships among body mass, flightlessness, and herbivory, and the potential importance of the avian feeding apparatus and foraging behaviors in explaining dietary evolution along body mass gradients in birds.

## Materials and Methods

### Diet data and indices

I compiled diet data from 208 quantitative studies and 11 qualitative studies (Appendix S1) to create a continuous character representing the extent of herbivory in 14 (of 287) species of Galliformes, the sister clade of Anseriformes, and 99 (of 168) species of Anseriformes (Clements et al. 2014). These 113 species represent 13 of the 76 galliform genera and 37 of the 52 anseriform genera. Galliform taxa, which were included to better circumscribe estimates for the ancestral dietary state of Anseriformes, were chosen based on those taxa for which the most detailed diet data were available and to include representatives of all five galliform families. I used handbooks (Johnsgard 1978; Marchant and Higgins 1990; Kear 2005; Poole 2005) and Google Scholar searches to locate the 219 primary diet data sources. When the primary source was not available, I took data from a secondary source. Appendix S1 lists the primary sources for all compiled diet data, including secondary sources, if applicable.

The data included in this meta‐analysis were collected using a wide range of methodologies, including the relative proportions of items in the gut (e.g., Landers et al. 1977) and the relative proportions of items in feces (e.g. Middleton and van der Valk 1987), and the percent of time animals were observed to spend feeding on particular foods (e.g. Naranjo 1986). Studies of gut contents varied in the portion of the gut from which items were removed (esophagus, crop, proventriculus, etc.) and the method used to assess the relative contribution of each item (aggregate percentage, percent volume, percent mass, frequency of occurrence, etc.). The particular method used and the portion of the gut from which items were removed, if applicable, are provided for each study in Appendix S1. Quantitative diet data were available for 106 (94%) of the 113 species included in this study; diets of the remaining seven species are known only by qualitative accounts. Qualitative accounts list items consumed by a particular species, verified either by visual observation of foraging behavior or gut contents, but without a numerical quantification of the relative contribution of each item to the diet. I excluded 69 species of Anseriformes owing either to insufficient diet data, a lack of body mass data, or uncertain phylogenetic position (i.e., lack of published molecular sequence data).

Avian diet data are often collected during a particular season or in a particular locality. When possible, I treated diet data from different seasons and localities as separate entries, totaling 403 entries. The total number of primary sources cited (219) is less than the number of entries as some sources contained data from multiple seasons, localities, or species. Thirty species are represented by a single entry, and the maximum number of entries for one species is 11. For each entry, both qualitative and quantitative, I grouped each food item consumed into one of eight nonoverlapping categories (Table 2). I calculated the relative importance of each category as a percentage such that all categories summed to 100% for each entry. For quantitative studies reporting the percentage of gut contents or percentage of feeding time, I used the raw percentages directly. For studies reporting the frequency of occurrence of gut or fecal contents, I normalized the relative frequencies such that the frequencies summed to 100%. Although qualitative studies do not quantify the relative importance of each item, importance is often indicated by descriptive adjectives (e.g., primarily leaves, rarely seeds). To take this into account, I used the qualitative descriptions to score items on a scale from 1 to 4, ranging from a rare to primary component of the diet. If no such description was given, I scored each item equally. I then normalized the scores such that all scores for an account summed to 100 to obtain a category sum in the form of a percentage. The number of specimens or individuals sampled, the season, locality, and the sums for each category and entry are provided in Appendix S1.

**Table 2:** Dietary categories used in this study and the corresponding parts and taxa included in that category. The herbivory index is the sum of the proportions of leaves, plants, roots, and algae. The folivory index is the sum of the proportions of leaves and plants

Category	Parts and taxa included
Leaves	Fibrous parts of plants: leaves, stems, root stalks, needles, branches of Embryophyta (“land plants”)
Roots	Roots, rhizomes, and bulbs of Embryophyta
Seeds	Seeds and nuts of Embryophyta
Fruits	Fruits, flowers, catkins, or spores of Embryophyta
Plants	Embryophyta, part is not specified (e.g. “15% dandelion”)
Algae	Chlorophyta or charophyceae (green algae), rhodophyta (red algae), phaeophyceae (brown algae), or cyanobacteria
Animal	Metazoa
Other	Any taxon not included in the above categories or unidentified matter

I averaged the percentage representation of each category across all entries for each species, giving equal weight to each entry. To calculate the extent of herbivory for each species, I summed the dietary categories that include the leaves and roots of plants and algae (Table 2) and divided by the sum of all categories except “other” (since this often included unidentified matter). This produces a value between 0 and 1, referred to here as herbivory index. Items identified as a plant but for which the part of the plant was not specified (“plant” category), are assumed in most cases to represent the fibrous parts of plants and are thus included in herbivory index. Most arguments concerning the potential correlation between diet and body mass are based on the relative digestibilities of dietary components. Because roots, rhizomes, and bulbs are slightly more digestible on average than leaves, stems, and buds (Karasov 1990), a second dietary index was also calculated summing only the leaves and unidentified plant parts. This is referred to here as folivory index and represents a diet that is a less digestible subset of the herbivory index. The mean diet category sums, herbivory index, and folivory index for each species are provided in Appendix S2. Prior to all correlation tests, I logit‐transformed both dietary indices to account for a skew toward small values, first changing all zeros and ones to the minimum and maximum values, respectively (Warton and Hui 2011; Sokal and Rohlf 2012). Logit transformation yielded approximately normal distributions of dietary indices (Appendix S3).

### Phylogenetic trees

To model the evolution of herbivory in Anseriformes and account for phylogenetic relatedness in testing for a correlation between herbivory and body mass, I used a published phylogeny of 6714 avian species, pruned to include the 113 galliform and anseriform species in this study (Burleigh et al. 2015). Constructed from a sparse supermatrix of 22 nuclear loci and seven mitochondrial regions, this tree represents the most current molecular phylogeny of galloanserae (the monophyletic clade comprising Galliformes and Anseriformes), incorporating data from several previous studies (Sraml et al. 1996; Johnson and Sorenson 1998; McCracken et al. 1999; Sorenson et al. 1999; Donne‐Goussé et al. 2002) and with branch lengths scaled to molecular sequence divergence. To account for uncertainty in topology and branch lengths, I performed all analyses across a sample of 100 maximum‐likelihood bootstrap trees obtained from the Dryad data repository (Burleigh et al. 2014). The tree was made ultrametric solely for visualization purposes using the “chronos” function in the ape package version 3.1‐4 (Paradis et al. 2004); all analyses were performed on nonultrametric trees.

### Body mass data

I obtained body masses for all species except the extinct moa‐nalo *Thambetochen chauliodous* from Dunning (2008), using species averages and pooling subspecies (*N *=* *1–7608; Appendices S1 and S2). Body masses are sexually dimorphic within the sampled taxa, with males weighing about 225 g on average more than females. However, because sex‐segregated data for body mass, and to a greater extent diet, are lacking for most sampled taxa, I took the arithmetic mean of female and male body masses. I obtained the mass of *T. chauliodous*, (6227.5 g) from an estimate by Iwaniuk et al. (2004) based on tibiotarsal circumference. Mean body masses ranged from 166.3 g in *Callipepla californica* (California Quail) to 11.1 kg in *Cygnus buccinator* (Trumpeter Swan) with a mean across all sampled taxa of 1.71 kg and a standard deviation of 1.81 kg. I log_10_‐transformed body mass averages prior to the correlation tests to account for a skew toward small body mass.

### Trait models

To choose the best model for the ancestral state reconstruction of herbivory and folivory index, I compared five models of trait evolution: a Brownian motion model (Felsenstein 1973), an Ornstein–Uhlenbeck (OU) model (Butler and King 2004), a lambda model, a kappa model, and a delta model (Pagel 1999). The Brownian motion model fits two parameters to the trait data: an initial trait value and a rate parameter (Felsenstein 1973). The remaining models estimate these same two parameters with an additional parameter to account for several potential scenarios such as attraction toward a particular trait value, covariance among traits that is only partially predicted by the phylogeny, a relationship between character divergence and speciation, and time‐dependent trait evolution (Felsenstein 1973; Butler and King 2004). I fit each model to both dietary indices using the “fitContinuous” function in the geiger package version 2.0.3 (Harmon et al. 2008) of R version 3.1.3 (R Core Team 2015) across all 100 trees in the sample distribution and used AICc scores to compare the fit of each model.

### Ancestral state reconstructions

I used the best fitting model (here the lambda model) to reconstruct the ancestral state of herbivory and folivory index for the 113 species dataset. I estimated the lambda parameter for each tree using “fitContinuous”. I then estimated the ancestral states using the “rescale” function in geiger and the “ace” function in the ape package (Paradis et al. 2004), with the restricted maximum‐likelihood (REML) method (Felsenstein 1973; Schluter et al. 1997). I performed ancestral state reconstruction for the 100 trees in the sample distribution using the lambda parameter specific to each tree. As I performed ancestral state reconstruction across multiple trees of differing topology, I only recorded estimates of ancestral states (and their corresponding 95% confidence intervals) at five strict consensus nodes (nodes A‐E in Fig. 3); the ancestors of these nodes are identical across all trees. I selected these five nodes from a strict consensus tree of all 100 trees containing only the species included in this study, constructed using the “consensus” function in the ape package (Appendix S4; Paradis et al. 2004). I plotted the distributions of ancestral state estimates and their corresponding 95% confidence intervals using the “density” function in R (R Core Team 2015), applying a Gaussian kernel to produce smooth probability distributions. The peak of each distribution (mode) was determined by the maximum kernel density. I plotted estimates at all nodes onto the maximum‐likelihood topology using the “plot.phylo” function in ape (Paradis et al. 2004).

### Nonphylogenetic and phylogenetic correlation tests

To test for a correlation between diet and body mass, I performed nonphylogenetic and phylogenetic correlation tests between log_10_ body mass and logit‐transformed dietary index values (herbivory and folivory index). For nonphylogenetic correlation tests, I performed a Pearson's correlation test using the “cor.test” function in the R stats package (R Core Team 2015). Major axis (MA) regression and standardized major axis regression, using the “lmodel2” function in the R package lmodel2 (Legendre 2014), were performed solely for trend line visualization.

Because mass and diet data were collected from interrelated taxa, the trait values do not represent independent data points. I quantified the strength of phylogenetic signal by calculating Blomberg's *K* and Pagel's *λ* for dietary indices and body mass using the “phylosig” function in the phytools package (Pagel 1999; Blomberg et al. 2003; Revell 2012). To account for phylogenetic nonindependence, I tested for a correlation between the standardized phylogenetic independent contrasts (PICs) of diet and body mass, assuming the evolution of the traits could be simulated by Brownian motion (Felsenstein 1985). Before performing correlational analyses, I tested for proper standardization of branch lengths by testing for a correlation between the standard deviation and absolute value of the contrasts (Garland et al. 1992). I used the “pearson” and “kendall” methods available in the “cor.test” function to identify any linear or nonlinear trends, respectively. If the standard deviation and absolute value of the contrasts showed a significant trend, the branch lengths were exponentially or log‐transformed using a range of constants until no significant trend was observed. This was performed for each trait and for each tree in the bootstrap distribution.

Once branch lengths were properly standardized, I computed the contrasts using the “pic” function in ape (Paradis et al. 2004) and tested for a correlation (Pearson's and Kendall's) between the contrasts using “cor.test”. PICs, as opposed to phylogenetic generalized least squares (PGLS) regression analysis, were used because a regression assumes that the independent (predictor) variable is measured without error (Sokal and Rohlf 2012), which is not appropriate for this data set. While PGLS allows for the fitting of several different evolutionary models while performing the regression, these models explain phylogenetic signal in the residuals (the fit of the tree to the regression) and do not relate directly to the test of whether the variables are significantly correlated (Freckleton et al. 2002; Revell 2010).

## Results

### Diet classification

**Figure 2:**
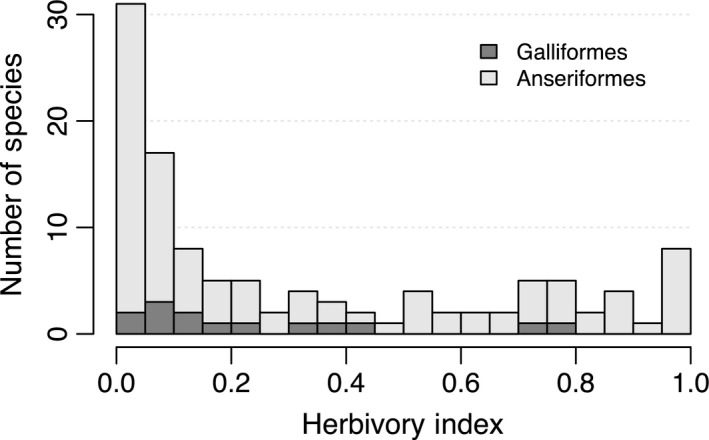
Histogram of herbivory index values for the Anseriformes (light) and Galliformes (dark) in this study.

**Figure 3:**
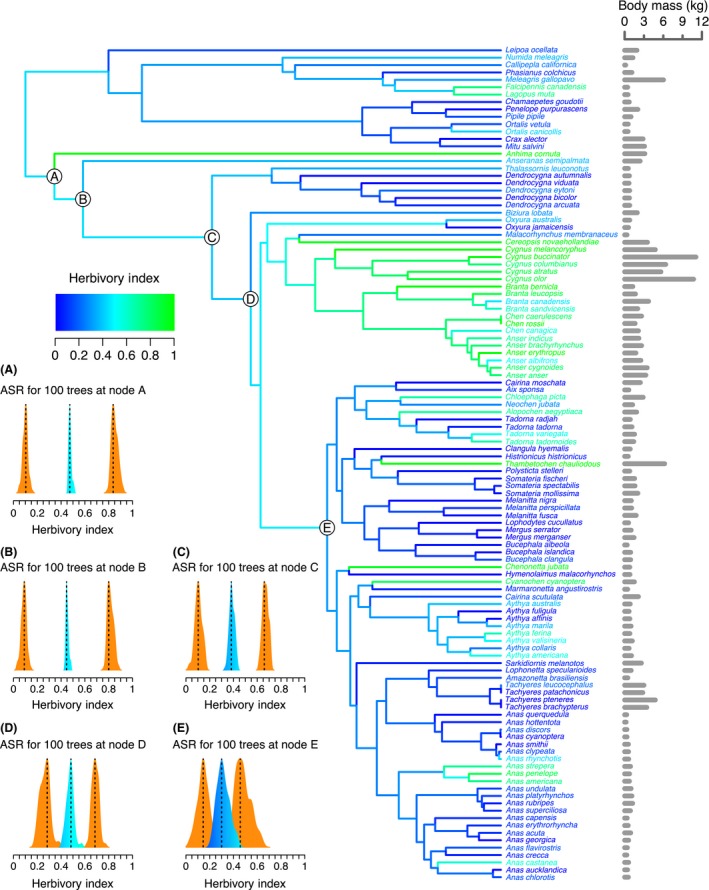
Ancestral state reconstruction of herbivory index in Anseriformes and a subset of Galliformes using a lambda model of trait evolution (Pagel 1999) on the maximum‐likelihood topology of Burleigh et al. (2015). Bars plotted to the right of each tip label show the mean body mass of that species in kilograms. Herbivory indices of each species are represented by branch tip and label colors and estimated ancestral values of herbivory index are represented by the color of internal branches, with green being most herbivorous and blue being least herbivorous. The results for the entire tree distribution (100 trees) at strict consensus nodes A‐E are summarized in the corresponding kernel density plots. For each consensus node, the distribution of ancestral herbivory estimates is shown in the same color scheme as the branches and the distribution of lower and upper 95% confidence intervals are shown in orange. Peaks of each distribution are indicated by dashed lines. Node A is the root of Anseriformes, node B is the root of Anatidae, and the sister clade to node A are the Galliformes.

Some degree of herbivory is widespread across galloanserae: of the taxa represented in this study, 71% of Anseriformes and 86% of Galliformes have an herbivory index greater than 5% (Fig. 2). However, several different galloanserae lineages exhibit a predominately herbivorous diet (Fig. 3). For example, I identified 25 anseriform species with an herbivory index greater than 70% (for a full table of values see Appendix S2). This includes species commonly called geese in the genera *Alopochen*,* Anser*,* Branta*,* Cereopsis*,* Chen*,* Chenonetta*, and *Cyanochen*. Also included are Horned Screamers (*Anhima cornuta*), all swans of the genus *Cygnus*, the moa‐nalo *T. chauliodous*, and three ducks of the genera *Anas*. Among the 14 Galliformes included in this study, two species, the Spruce Grouse (*Falcipennis canadensis*) and Rock Ptarmigan (*Lagopus muta*) also have predominately herbivorous diets, 76 and 74%, respectively. The results of dietary categorization using the more restricted folivory index are similar to the results with herbivory index and are not described further here but are provided in the supporting information.

### Trait models and ancestral state reconstruction

Of the five different trait models tested for the evolution of herbivory index, the lambda model is the best fitting model, having the lowest AICc score for 78% of the sample tree distribution (*N *=* *100; Appendix S5). Of these models, the lambda model also has the least variable AICc score across all topologies with a mean AICc score of 25.7 and a standard deviation of 3.4. The remaining 22% of trees are best fit by the kappa model, with a mean AICc score of 28.7 (SD = 5.1). The results of ancestral state reconstructions of herbivory index using the lambda model across the entire tree distribution (100 trees) are summarized in Figure 3. Estimates of the ancestral herbivory index at the five chosen consensus nodes range from 21 to 58% across all topologies. At the two deepest consensus nodes, the root of Anseriformes and the root of Anatidae (nodes A and B in Fig. 3, respectively; Clements et al. 2014), estimates of ancestral herbivory range from 42 to 52% but with 95% confidence intervals ranging from 3 to 92%, indicating the unreliability of these estimates. At the root node of all Anseriformes except *Anhima* and *Anseranas* (node C in Fig. 3), ancestral herbivory estimates range from 33 to 44% and upper 95% confidence intervals do not exceed 72%. At the most recent consensus node (E in Fig. 3), ancestral herbivory estimates range from 21 to 41% and upper 95% confidence intervals do not exceed 64%.

### Correlation tests

**Figure 4:**
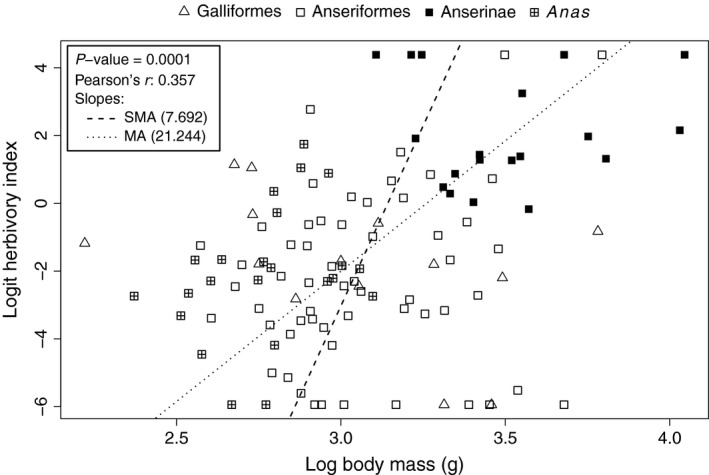
Plot of logit‐transformed herbivory index vs. log_10_ body mass for Anseriformes (square symbols) and a subset of Galliformes (triangle symbols). Two major clades within Anseriformes are indicated by different square symbols: filled squares for Anserinae (including the genera *Cereopsis*,* Cygnus*,* Anser*,* Branta*, and *Chen*) and crossed‐squares for the genus *Anas*. Significance statistics of a nonphylogenetic Pearson correlation test and slopes of nonphylogenetic major axis regressions are shown.

A nonphylogenetic correlation test identifies a significant relationship between body mass and herbivory index (*P *=* *0.0001; Fig. 4) although there is considerable scatter in this relationship (Pearson's *r* = 0.36). The trend is dominated by the Anserinae (which here includes the genera *Cereopsis*,* Cygnus*,* Branta*,* Chen*, and *Anser*) and *Anas* clades. Running counter to this trend are the steamer ducks (*Tachyeres*), which have large body masses and herbivory indices of 0–20%. Exclusion of the four species in *Tachyeres* increased the linearity of the trend (Pearson's *r* = 0.45). Galliformes also appear to oppose the positive trend between body mass and herbivory and exclusion of Galliformes (flightless steamer ducks not excluded) also increased the linearity of the trend (Pearson's *r* = 0.45).

**Figure 5:**
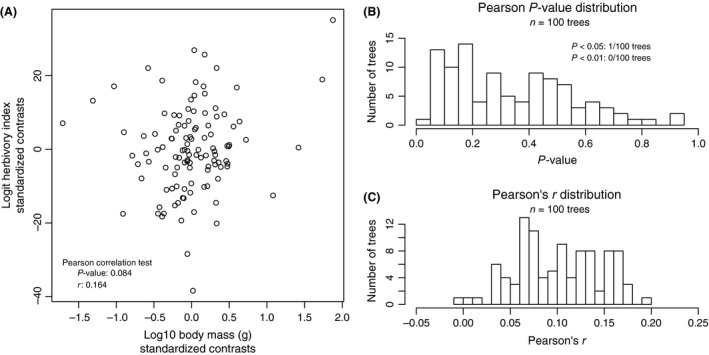
Pearson's correlation test on the standardized contrasts of logit‐transformed herbivory index and log_10_ body mass. (A) Plot of the contrasts for the maximum‐likelihood topology (Burleigh et al. 2015) and associated correlation statistics. Histogram of the *P*‐values (B) and Pearson's *r* (C) resulting from correlation tests performed across the entire tree sample (*n *=* *100). In (B), the number of trees that yielded *P*‐values less than 0.05 and 0.01 are indicated.

For all trees, significant phylogenetic signal was found in log_10_ body mass (*λ *= 0.98–0.99, *P *<* *0.001; *K* = 0.286–0.527, *P *<* *0.001) and logit herbivory index (*λ *= 0.73–0.86, *P *<* *0.001; *K* = 0.12–0.24, *P *<* *0.01), consistent with previously reported values for phylogenetic signal of ecological traits in birds (Smith 2012). The full phylogenetic signal results are reported in Appendix S8. Pearson's and Kendall's correlation tests between the standardized independent contrasts of body mass and herbivory recovered a significant relationship at the *α =* 0.05 threshold for only 1% of trees and did not recover a significant relationship at the *α =* 0.01 threshold for any trees (Fig. 5A,B). Correspondingly, values of Pearson's *r* were low, ranging from −0.01 to 0.19 for all trees (Fig. 5C). The full results of branch length transformations, standardization correlation tests, and correlation tests between body mass and herbivory index are detailed in Appendix S10.

## Discussion

Compilation of diet data from 219 primary sources reveals that some degree of herbivory is widespread across galloanserae with several independent lineages exhibiting a predominately herbivorous diet. Such distributed and wide‐ranging degrees of herbivory necessarily limit the potential of evolutionary trait models to confidently estimate ancestral states and, as expected, the deepest consensus nodes of the tree are characterized by wide confidence intervals. However, at consensus nodes with narrower confidence intervals, ancestral state reconstruction consistently favors an ancestor with a less herbivorous diet, with estimates ranging from 21 to 58%. In particular, ancestral estimates at the most recent consensus node within Anseriformes considered in this study support at least five independent increases to a predominately herbivorous diet from an ancestor that was likely less than 45% herbivorous. Correlation tests using phylogenetic independent contrasts show that the apparently significant positive correlation between herbivory and body mass is driven primarily by a couple of large clades. In the following discussion, I will consider limitations and assumptions in the interpretation of these results as well as explore the implications of these results for the origins of geese and the evolution of herbivory more broadly across birds.

### Diet as a continuous character

With over 200 published primary sources, we know more about the diets of Anseriformes than perhaps any other order of birds, both in terms of the proportion of species studied and the quantitative nature of the data. To my knowledge, this study represents the most comprehensive compilation to date of avian diet data from the literature applied to the generation of continuous dietary characters, ancestral state reconstruction, or correlation with other ecological characters. However, in representing this wealth of data as a single continuous ecological character, I have made several simplifying assumptions. Firstly, the studies from which I compiled these data weigh the relative contributions of food items using different methods (e.g., by mass, volume, frequency of occurrence), which is known to introduce biases when comparing among studies (Swanson and Bartonek 1970; Swanson et al. 1974). By normalizing the contribution of each dietary component for each study, I sought to generate an ecological character that represents the relative importance of herbivorous or folivorous food items in the broadest sense for each species.

Secondly, anseriform diets can vary considerably between seasons and localities. For example, the diet of Canvasbacks (*Aythya valisineria*) consists of 95% animal matter during the winter in Chesapeake Bay (Perry and Uhler 1988) but shifts to 99% fibrous plant material during fall migration in Wisconsin (Korschgen et al. 1988). By taking an average across several studies (when available), each dietary index represents the relative importance of the relevant food items across seasons and localities (e.g., species that are herbivorous throughout the year have a higher herbivory index than those with an herbivorous diet for only a portion of the year).

Thirdly, I have also not accounted for any potential geographic sampling biases. Geography could introduce a bias particularly for migratory Anseriformes. Diets are better known for more accessible nonbreeding grounds relative to less‐accessible breeding grounds. For example, of the nine entries included in this meta‐analysis to describe the diet of the Greater White‐fronted Goose (*Anser albifrons*), eight document the diet on nonbreeding grounds, while one documents the diet on breeding grounds (Appendix S1). In the *Anser* genus, for example, diets tend to be more herbivorous, by approximately 10%, on breeding grounds relative to nonbreeding grounds. But time spent on breeding grounds may only account for one quarter of the year. Thus, lack of correction in such cases may result in a slight underestimate of the extent of herbivory. Despite these limitations, the use of a continuous character to describe an organism's diet represents a significant advance relative to the traditional use of discrete characters: it more closely corresponds to the biological reality of a diverse character that often defies discrete categorization and it allows for direct comparisons with other continuous ecological characters (e.g., body mass, beak shape).

### The evolution of herbivory in Anseriformes and the origins of geese

Most Anseriformes have been given common names that classify them as either a “duck” or “goose”. Such a distinction is not based on any definite or discrete character, neither ducks nor geese form a monophyletic clade, and some species are even referred to as both duck and goose (e.g., Australian wood duck or Maned goose, *Chenonetta jubata*). Yet “duck” and “goose” do capture unmistakable characteristics that are distinguishable across the broad spectrum of anseriform beak shapes and foraging behaviors. Ducks generally have flatter, broader, and longer beaks and feed primarily in water, by dabbling or diving, while geese have taller, shorter beaks and feed primarily on land by grazing and digging (Johnsgard 1978). The classification of diets across Anseriformes compiled here supports a general distinction based on herbivory: geese tend to be among the most herbivorous Anseriformes, although notable exceptions include more aquatic herbivores such as swans and herbivorous ducks in the genera *Anas* and *Aythya* (Fig. 3). Considering this, geese are perhaps best thought of as Anseriformes possessing a collection of characters at the extremes of terrestriality and herbivory relative to other members of the order. Thus, the evolution of herbivory in Anseriformes yields important insights into the origins of geese, within the broader context of dietary evolution in Anseriformes.

Speculation on the origin of geese spans nearly a century and a half. Darwin (1872, p. 183) was perhaps the first, supposing that the finer, more numerous lamellae of ducks evolved from an ancestral condition of coarser, less numerous lamellae observed among several geese today, a “goose‐to‐duck” transition. The potential phylogenetic affinity of Anseriformes to an order of aquatic shorebirds (Delacour and Mayr 1945; Sibley et al. 1969) and the discovery of the stem anseriform fossil *Presbyornis* (Olson and Feduccia 1980), with an undeniably duck‐like skull and wader‐like postcranial skeleton, introduced the possibility that the transition proceeded in the exact opposite direction, from duck to goose. However, from recent phylogenetic studies, a clear sister relationship between Anseriformes and the exclusively terrestrial Galliformes has emerged (Sibley et al. 1988; Hackett et al. 2008), seemingly supporting a terrestrial feeding ecology as ancestral for Anseriformes and tipping the balance of evidence back in favor of the goose‐to‐duck hypothesis. The ancestral state reconstruction of diet presented in this study is unable to resolve the polarity of this transition by reliably estimating the diet at the root of Anseriformes (Fig. 3). However, at a more recent node, there is sufficient resolution to support several independent increases in herbivory from a primarily nonherbivorous ancestor (node E in Fig. 3). This establishes a duck‐to‐goose polarity for multiple transitions giving rise to geese in the genera *Chloephaga*,* Alopochen, Thambetochen*,* Chenonetta*, and *Cyanochen*.

Given the remarkably convergent characteristics of geese, it is likely that a similar pattern of duck‐to‐goose transitions has given rise to additional lineages of geese throughout the evolutionary history of Anseriformes, including the Cape Barren Goose (*Cereopsis novaehollandiae*) and geese in the genera *Anser*,* Branta* and *Chen*.

Two additional lines of evidence support a duck‐to‐goose transition. The first is the near ubiquity of lamellae among Anseriformes. Lamellae are keratinous comb‐like ridges that line the upper and lower bills of nearly all Anseriformes. The only Anseriformes lacking lamellae are the mergansers (*Mergus* and *Lophodytes*), which instead have keratinous, tooth‐like serrations, and the screamers (*Anhima* and *Chauna*), which may in fact possess vestigial lamellae (Olson and Feduccia 1980). Although lamellae could function in stripping seeds or grasping vegetation (van der Leeuw et al. 2003), they likely evolved originally for filter‐feeding. The only other avian lineages possessing comparable structures, flamingos and prions, also use lamellae to filter‐feed (Klages and Cooper 1992; Zweers et al. 1995). In vivo experiments have demonstrated a trade‐off in performance between filter‐feeding and grazing such that specialization in one is accompanied by decreased performance in the other (van der Leeuw et al. 2003). Such a trade‐off and the widespread distribution of lamellae among Anseriformes support the hypothesis that the evolution of grazing, and therefore herbivory, represents a shift away from more duck‐like, primarily filter‐feeding ancestor. The second line of evidence is the Lower Eocene fossil anseriform *Presbyornis* (Olson and Feduccia 1980), which has a three‐dimensionally preserved beak remarkably similar to the modern Freckled Duck (*Stictonetta naevosa*) or Pink‐eared Duck (*Malacorhynchus membranaceus*). In spite of a radically non‐duck‐like postcranial skeleton, this fossil, commonly placed at the stem of the sister group to *Anseranas* (node C in Fig. 3; Livezey 1997; Clarke et al. 2005), establishes a definitively duck‐shaped bill early in the history of Anseriformes in support of the duck‐to‐goose hypothesis.

These results are also consistent with the proposed theory for the origin of the moa‐nalos, a lineage of recently extinct herbivorous geese in the Hawaiian Islands (Olson and James 1991). The skulls of the moa‐nalos bear a strong resemblance to those of the exclusively herbivorous Cape Barren Goose of southern Australia and evidence from coprolites confirms that moa‐nalos likely fed exclusively on the leaves of native vegetation (James and Burney 1997). Yet despite several goose‐like features, morphological and molecular data from subfossils place moa‐nalos as an early divergence within the subfamily of dabbling ducks Anatidae and not closely related to any extant lineages of geese (Olson and James 1991; Sorenson et al. 1999). Thus, it has been proposed that moa‐nalos evolved from a filter‐feeding duck within the past 6 million years, the age of the oldest Hawaiian Island, Kauai (Sorenson et al. 1999). The results of this study support this theory by not only estimating a low degree of herbivory as ancestral for the clade from which moa‐nalos are most likely to have evolved, but also by identifying moa‐nalos as one example of a series of parallel shifts toward increased herbivory in the evolutionary history of Anseriformes.

### The correlation between herbivory and body mass in birds

The historic expectation that body mass is positively correlated with a more herbivorous diet in birds (Morton 1978; Dudley and Vermeij 1992; Klasing 1998) likely stems from a classic “digestive efficiency” hypothesis that a larger body mass provides advantages in the use of low‐quality diets (Geist 1974; reviewed in Clauss et al. 2013). Such a hypothesis invokes a physiological mechanism whereby differential scaling of digestive parameters, such as food intake rate, causes larger herbivores to outcompete smaller herbivores or increase the herbivorous portion of their diet. The “abundance‐packet size” hypothesis proposes an ecological mechanism whereby increases in body mass, for reasons perhaps unrelated to dietary shifts, causes animals to shift toward diets composed of foods that are available in increasingly abundant or large packets (Hiiemae 2000; Clauss et al. 2013). In the spectrum of food abundance and packet size, it is supposed that leaves of plants and large animals lie at the extremes of abundance and packet size, respectively. This leads to the prediction that a shift toward larger body mass in noncarnivorous lineages would lead to an increase in herbivory. While both of these hypotheses predict correlated increases between herbivory and body mass, they differ in the proposed sequence of increases: the digestive efficiency hypothesis predicts that increases in body mass follow shifts toward herbivory, while the abundance‐packet size hypothesis predicts that shifts toward herbivory follow increases in body mass.

This is the first study to directly test the relationship between body mass and an herbivorous diet in birds in a phylogenetic context. The data show that while there are several predominately herbivorous Anseriformes of large body mass, these are primarily clustered in a single clade (Figs 3 and 4). Additionally, there are also herbivorous Anseriformes of small body mass which represent independent shifts toward increased herbivory: The American Wigeon (*Anas americana*), Blue‐winged goose (*Cyanochen cyanoptera*), and Australian Wood Duck (*Chenonetta jubata*) are all predominately herbivorous but have average body masses between 750 and 1500 g. Within the Galliformes, the predominately herbivorous Rock Ptarmigan (*Lagopus muta*) and Spruce Grouse (*Dendragapus canadensis*) also have low average body masses relative to both Anseriformes and other Galliformes, less than 600 g (Dunning 2008). Thus, once phylogeny is taken into account and for a range of assumed phylogenetic relationships, the apparently significant correlation between herbivory and body mass observed in a nonphylogenetic correlation test is no longer significant (Fig. 5).

The results of this study do not support the digestive efficiency hypothesis: independent transitions toward increased herbivory are not consistently associated with an increase in body mass. Other vertebrate groups, such as lizards (Pough 1973; Schluter 1984; Cooper and Vitt 2002) and terrestrial mammals (Price and Hopkins 2015), do exhibit a correlation between herbivory and larger body mass. Yet recent studies have shown that this association is not explained by increased digestive efficiency. Studies in mammals, lizards, and birds investigating the relationship between food intake rate and body mass and between mean retention time and body mass, the two primary mechanisms by which the digestive efficiency is proposed to increase have found little or no evidence that an increase in body mass per se confers a digestive advantage (Clauss et al. 2008; Franz et al. 2011; Fritz et al. 2012; Müller et al. 2013; Steuer et al. 2014). Thus, this study contributes to a growing body of literature refuting a physiological link between body mass and herbivory or dietary digestibility more generally.

The results of this study also appear to contradict the abundance‐packet size hypothesis that increases in body mass are associated with shifts toward herbivory. However, two caveats may preclude this study from drawing a strong conclusion regarding this hypothesis. First, the abundance‐packet size hypothesis predicts that increases in body mass are followed by increased herbivory. Within Anseriformes, there are likely only three pronounced increases in body mass: the Anserinae, the moa‐nalos (represented by *Thambetochen*), and the steamer ducks (*Tachyeres*). The Anserinae and moa‐nalos conform to the ecological prediction, showing increases in body mass and shifts to predominately herbivorous diets, while steamer ducks have evolved molluscivory as their predominate feeding mode (Weller 1972; Agüero et al. 2014). As these two hypotheses make different predictions regarding the sequence of changes in body mass and diet, there may be a sufficient number of dietary shifts to robustly test the digestive efficiency hypothesis, but an insufficient number of body mass changes to robustly test the abundance‐packet size hypothesis.

The second caveat that may preclude using these results to make a strong conclusion regarding the abundance‐packet size hypothesis is that the entire order Anseriformes, relative to other birds, may in fact lie at the extreme of the ecological body mass‐diet spectrum, characterized by large body masses and abundant or large packet food sources. In contrast to browsing or catching flying insects on the wing, filter‐feeding enables Anseriformes to collect large quantities of aquatic insect larvae, bivalves, and seeds en masse (Kooloos et al. 1989; van der Leeuw et al. 2003; Gurd 2006). Piscivory and molluscivory, in *Mergus* and *Tachyeres*, for example, enable the acquisition of large packets of animal food, in analogy to carnivory. And although body masses within this sample span two orders of magnitude, the sampled taxa are heavy relative to other birds: the lightest species in this study (*C. californica*, 166 g) is heavier than 78% of extant birds (Dunning 2008). Thus, there may also be insufficient variation in food source abundance or packet size to test the abundance‐packet size hypothesis.

Even if Anseriformes occupied a wider range of the body mass‐diet spectrum to robustly test the abundance‐packet size hypothesis, it is unlikely that a body mass‐diet spectrum, as it is currently framed, can adequately explain dietary evolution in birds. Although a seed‐eating duck and seed‐eating passerine both have diets composed primarily of seeds, the packet sizes differ considerably because of differences between filter‐feeding and browsing among individual seed pods and plants. Foraging behavior provides a key link between diet and packet size and likely relates more directly to body mass than diet per se. Thus, a body mass‐foraging behavior spectrum may prove more useful in explaining dietary variation along body mass gradients in birds than a body mass‐diet spectrum.

### Exceptional avian herbivores

If herbivory in birds is not necessarily constrained by an increase in body mass, why is herbivory not more widespread in birds? And what accounts for the exceptional number of transitions toward increased herbivory in Anseriformes relative to other bird orders? An herbivorous diet is a considerable digestive challenge, owing to the low digestibility of leaves, stems, and underground parts of plants relative to other foods commonly consumed by birds (Karasov 1990). Herbivorous Anseriformes appear to compensate for this low digestibility by adopting a “high‐throughput” strategy: high food intake and short mean retention time (Clauss et al. 2007; Fritz et al. 2012; Frei et al. 2014). It has been observed more generally across Anseriformes that as the quality of the food ingested increases, less time is spent engaged in food intake both across species (Paulus 1988) and as a response to seasonal shifts within species (Paulus 1984; Brodsky and Weatherhead 1985). As a consequence of higher ingestion rates, mean retention times in herbivorous Anseriformes are shorter by an order of magnitude than avian herbivores that spend less time feeding and rely more on gut fermentation such as the Hoatzin or ostriches (Grajal et al. 1989; Karasov 1990; Mayhew and Houston 1993; Fritz et al. 2012).

This high‐throughput strategy poses its own challenges and requires that herbivorous Anseriformes graze continuously for several hours, in some cases over 9 h per day (Owen 1972; Ebbinge et al. 1975). Grazing involves cyclical movements of the upper and lower beak to crop the leaves of plants and the tongue to transport food items into the pharynx (van der Leeuw et al. 2003). This continuous and cyclical motion of the beak is fundamentally different from the way in which most other birds use their beak to feed. Surprisingly, filter‐feeding is perhaps the most similar beak behavior among birds to grazing; the upper and lower beak also open and close cyclically in coordination with the tongue to serve as a suction pump for fluid (Zweers et al. 1995; van der Leeuw et al. 2003). Although there is a trade‐off in performance between grazing and filter‐feeding in Anseriformes, they are not mutually exclusive behaviors and many grazers continue to use filter‐feeding to some degree (Johnsgard 1978; van der Leeuw et al. 2003).

While it is perhaps tempting to speculate that filter‐feeding, with its similarities to the demands of grazing and likely ancestral distribution among Anseriformes, has facilitated the repeated evolution of herbivory in Anseriformes, empirical support for this hypothesis is lacking. Filter‐feeding has evolved two other times among birds, in flamingos and prions (Klages and Cooper 1992; Zweers et al. 1995), yet neither of these clades displays any tendency toward herbivory (although the mechanism of filter‐feeding and ecological context also vary considerably). Additionally, emus have also evolved a high‐throughput strategy to herbivory, in contrast to the independently evolved, low‐throughput strategy of ostriches, indicating such a strategy is not unique to Anseriformes (Frei et al. 2014).

Anseriformes are also exceptional, relative to other birds, in that transitions to increasing herbivory have occurred, with the exception of the moa‐nalos, without the loss of flight. Outside of Anseriformes, herbivory tends to occur in birds that have lost flight or show lower flight capacity (Table 1). Several of these lineages are island endemics: the elephant birds of Madagascar (Clarke et al. 2006) and the Moas, Takahe, and Kakapo of New Zealand (Mills and Mark 1977; Trewick 1996; Wood et al. 2008). Although island endemism is not a direct cause of increased body mass in birds (Gaston and Blackburn 1995), it is a driving factor in the loss of flight (McNab 1994) which then lessens constraints on a subsequent increase in body mass. Given the result of this and prior studies, the digestive efficiency hypothesis is unlikely to explain the apparent associations among body mass, flightlessness, and herbivory outside of Anseriformes. However, the results of this study do not exclude the possibility that the abundance‐packet size hypothesis could account for increasing herbivory in large‐bodied and flightless birds. Indeed, flightlessness may be an additional factor that, independent of increases in body mass, could drive shifts toward an abundant and easily accessible food source such as herbivory.

Although the underlying causes may remain unclear, the repeated evolution of a more herbivorous diet and its lack of association with a higher body mass or loss of flight in Anseriformes prompt a reinterpretation of the relatively infrequent origination of herbivory among flighted birds. The unique and versatile feeding apparatus of Anseriformes has likely played a major role in the dietary evolution of this clade. Comparisons of the foraging behaviors, in vivo mechanics and morphology of the feeding apparatus in different lineages of filter‐feeding and grazing Anseriformes will shed important insights on the relationships between feeding morphology and diet, the functional analogies between these feeding strategies and the remarkable number of dietary transitions that characterize this diverse clade.

## Conflict of Interest

None declared.

## Supporting information


**Appendix S1.** Excel spreadsheet with the complete diet and body mass (from Dunning 2008) dataset, including associated metadata, listed by species, season and locality.

**Appendix S2.** Excel spreadsheet containing the mean dietary category sums, dietary indices and body masses for each species in Appendix S1 (compact version of Appendix S1).

**Appendix S3.** Histograms and Q‐Q plots showing approximate normalization of diet trait values (herbivory and folivory indices) using logit‐transformation.
**Appendix S4.** Strict consensus tree for the entire sample tree distribution (100 trees) from Burleigh et al. 2015, pruned to include only taxa in this study.
**Appendix S5.** AICc scores applying five different models of trait evolution to herbivory index and folivory index across the tree distribution (100 trees).
**Appendix S6.** Ancestral state reconstruction of folivory index (folivory equivalent to Figure 3).
**Appendix S7.** Non‐phylogenetic correlation of logit‐transformed folivory index and log_10_ body mass (folivory equivalent to Figure 4).
**Appendix S8.** Ranges of phylogenetic signal in herbivory index and body mass.
**Appendix S9.** Correlation of folivory index and body mass contrasts (folivory equivalent to Figure 5).
**Appendix S10.** Phylogenetic independent contrast results, correlation coefficients and branch length transformations.
**Appendix S11.** Literature cited in diet meta‐analysis (primary and secondary sources in Appendix S1).
