# Supplementary material for: Exceptional avian herbivores: multiple transitions toward herbivory in the bird order Anseriformes and its correlation with body mass
Source: Ecol Evol. 2015 Oct 15;5(21):5016–32. doi: 10.1002/ece3.1787 (PMC4662324; doi:10.1002/ece3.1787)
Supplement: Supplementary file 3 — Appendix S3. Histograms and Q‐Q plots showing approximate normalization of diet trait values (herbivory and folivory indices) using logit‐transformation. Appendix S4. Strict consensus tree for the entire sample tree distribution (100 trees) from Burleigh et al. 2015, pruned to include only taxa in this study. Appendix S5. AICc scores applying five different models of trait evolution to herbivory index and folivory index across the tree distribution (100 trees). Appendix S6. Ancestral state reconstruction of folivory index (folivory equivalent to Figure 3). Appendix S7. Non‐phylogenetic correlation of logit‐transformed folivory index and log10 body mass (folivory equivalent to Figure 4). Appendix S8. Ranges of phylogenetic signal in herbivory index and body mass. Appendix S9. Correlation of folivory index and body mass contrasts (folivory equivalent to Figure 5). Appendix S10. Phylogenetic independent contrast results, correlation coefficients and branch length transformations. Appendix S11. Literature cited in diet meta‐analysis (primary and secondary sources in Appendix S1). [file ECE3-5-5016-s003.docx]

**Appendix**

**Appendix S1** ......................................................... See accompanying excel file **Appendix S1.xls**

Excel spreadsheet with the complete diet and body mass (from Dunning 2008) dataset, including associated metadata, listed by species, season and locality.

**Appendix S2** ......................................................... See accompanying excel file **Appendix S2.xls**

Excel spreadsheet containing the mean dietary category sums, dietary indices and body masses for each species in Appendix S1 (compact version of Appendix S1).

**Appendix S3** .............................................................................................................................. 2-3

Histograms and Q-Q plots showing approximate normalization of diet trait values (herbivory and folivory indices) using logit-transformation.

**Appendix S4** .................................................................................................................................. 4

Strict consensus tree for the entire sample tree distribution (100 trees) from Burleigh et al. 2015, pruned to include only taxa in this study.

**Appendix S5** .................................................................................................................................. 5

AICc scores applying five different models of trait evolution to herbivory index and folivory index across the tree distribution (100 trees).

**Appendix S6** .................................................................................................................................. 6

Ancestral state reconstruction of folivory index (folivory equivalent to Figure 3).

**Appendix S7** .................................................................................................................................. 7

Non-phylogenetic correlation of logit-transformed folivory index and log_10_ body mass (folivory equivalent to Figure 4).

**Appendix S8** .................................................................................................................................. 8

Ranges of phylogenetic signal in herbivory index and body mass.

**Appendix S9** .................................................................................................................................. 9

Correlation of folivory index and body mass contrasts (folivory equivalent to Figure 5).

**Appendix S10** ..................................................... See accompanying excel file **Appendix S10.xls**

Phylogenetic independent contrast results, correlation coefficients and branch length transformations.

**Appendix S11** ........................................................................................................................ 10-23

Literature cited in diet meta-analysis (primary and secondary sources in Appendix S1)

**Appendix S3.** Histograms and Q-Q plots showing approximate normalization of diet trait values using logit-transformation. HI indicates herbivory index, FI (next page) indicates folivory index.

**Appendix S3.** (continued)

**Appendix S4.** Strict consensus tree for the entire sample tree distribution (100 trees) from Burleigh et al. 2015, pruned to include only taxa in this study.


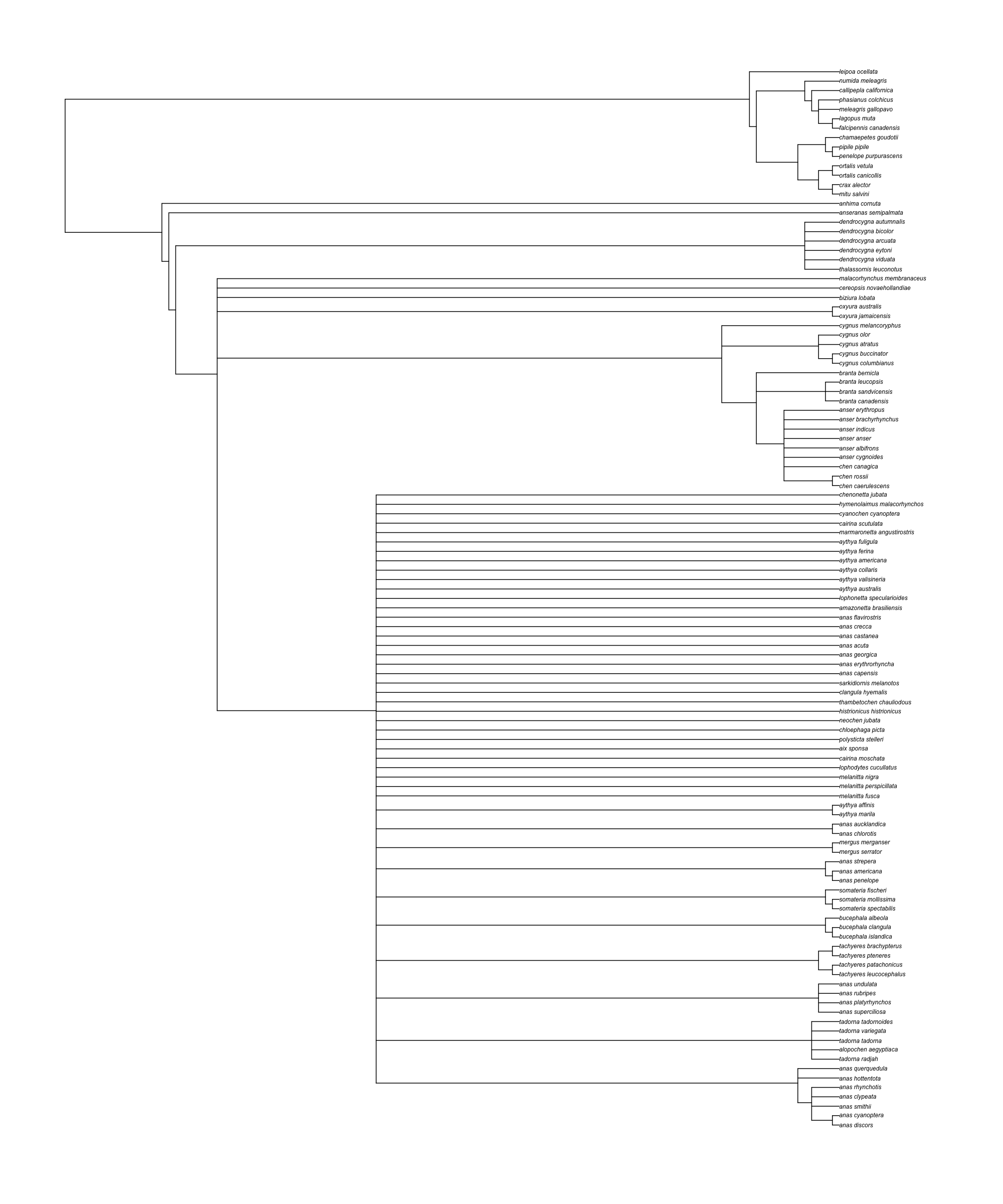


**Appendix S5.** AICc scores applying five different models of trait evolution to herbivory index (top) and folivory index (bottom) across the tree distribution (100 trees).

**Appendix S6.** Ancestral state reconstruction of folivory index (folivory equivalent to Figure 3).

**Appendix S7.** Non-phylogenetic correlation of logit-transformed folivory index on log_10_ body mass (folivory equivalent to Figure 4).

**Appendix S8.** Ranges of phylogenetic signal in herbivory index and body mass across the sample of 100 trees. A small number of short branch lengths caused the calculation of Blomberg’s K to be low and non-significant (no such effect was observed for Pagel’s λ). This is interpreted as a sensitivity of Blomberg’s K to very short branch lengths. Setting branch lengths less than two times the minimum branch length to the median branch length was sufficient to cause Blomberg’s K to yield values consistent with previous studies testing phylogenetic signal in body mass and ecological traits (Smith 2012).

|  | **Pagel’s λ** | | | |
| --- | --- | --- | --- | --- |
|  | **λ** | | ***p*-value** | |
|  | **min** | **max** | **min** | **max** |
| Logit herbivory index | 0.730 | 0.860 | <0.0001 | <0.0001 |
| Log_10_ body mass | 0.975 | 0.999 | <0.0001 | <0.0001 |

|  | **Blomberg’s Κ** | | | |
| --- | --- | --- | --- | --- |
|  | **K** | | ***p*-value** | |
|  | **min** | **max** | **min** | **max** |
| Logit herbivory index | 0.123 | 0.239 | 0.001 | 0.008 |
| Log_10_ body mass | 0.286 | 0.517 | 0.001 | 0.001 |

**Appendix S9.** Correlation of logit-transformed folivory index and log_10_ body mass contrasts (folivory equivalent to Figure 5).

**Appendix S11.** Literature cited in diet meta-analysis (primary and secondary sources in Appendix S1).

1. Afton, A. D., R. H. Hier, and S. L. Paulus. 1991. Lesser scaup diets during migration and winter in the Mississippi flyway. Can. J. Zool. 69:328-333.
2. Agüero, M. L., P. G. Borboroglu, and D. Esler. 2014. Trophic Ecology of Breeding White-Headed Steamer-Duck (*Tachyeres leucocephalus*). Waterbirds. 37:88-93.
3. Alexander, G. D. 2002. Observations of the endangered Trinidad Piping-Guan (*Pipile pipile*), or Pawi, in northern Trinidad. Studies in Trinidad and Tobago Ornithology Honouring Richard F French (FE Hayes and SA Temple, Eds.). Dept. Life Sci., Univ. West Indies, St. Augustine, Occ. Pap. 11:119-130.
4. Alisauskas, R. T., C. D. Ankney, and E. E. Klaas. 1988. Winter diets and nutrition of midcontinental lesser snow geese. J. Wildl. Manag. 52:403-414.
5. Amat, J. A. 1986. Some aspects of the foraging ecology of a wintering Greylag Goose *Anser anser* population. Bird Study. 33:74-80.
6. Anthony, R. G. 1970. Food habits of California quail in southeastern Washington during the breeding season. J. Wildl. Manag. 34:950-953.
7. Austin, J. E., J. R. Serie, and J. H. Noyes. 1990. Diet of canvasbacks during breeding. Prairie Nat. 22:171-176.
8. Ayeni, J. S. O. 1983. The biology and utilization of helmeted guinea-fowl (*Numida meleagris galeata* Pallas) in Nigeria. II. Food of helmeted guinea-fowl in Kainji Lake Basin area of Nigeria. Afr. J. Ecol. 21:1-10.
9. Badzinski, S. S., and S. A. Petrie. 2006. Diets of lesser and greater scaup during autumn and spring on the lower Great Lakes. Wildl. Soc. Bull. 34:664-674.
10. Bailey, M., S. A. Petrie, and S. S. Badzinski. 2008. Diet of mute swans in lower Great Lakes coastal marshes. J. Wildl. Manag. 72:726-732.
11. Baldwin, J. R., and J. R. Lovvorn. 1994. Habitats and tidal accessibility of the marine foods of dabbling ducks and brant in Boundary Bay, British Columbia. Mar. Biol. (Berl.). 120:627-638.
12. Bartonek, J. C., and J. J. Hickey. 1969. Food habits of canvasbacks, redheads, and lesser scaup in Manitoba. Condor. 71:280-290.
13. Bergman, R. D. 1973. Use of southern boreal lakes by postbreeding canvasbacks and redheads. J. Wildl. Manag. 37:160-170.
14. Bisset, S. A. 1976. Foods of the Paradise Shelduck *Tadorna variegata* in the high country of North Canterbury, New Zealand. Notornis. 23:106-119.
15. Black, J. M., J. Prop, J. M. Hunter, F. Woog, A. P. Marshall, and J. M. Bowler. 1994. Foraging behaviour and energetics of the Hawaiian goose *Branta sandvicensis*. Wildfowl. 45:65-109.
16. Bolen, E. G., and B. J. Forsyth. 1967. Foods of the Black-bellied Tree Duck in south Texas. Wilson Bull. 79:43-49.
17. Booth, D. T. 1986. Crop and gizzard contents of two Mallee Fowl. Emu. 86:51-53.
18. Botero, J. E., and D. H. Rusch. 1994. Foods of blue-winged teal in two neotropical wetlands. J. Wildl. Manag. 58:561-565.
19. Bourne, G. R. 1981. Food habits of black-bellied whistling ducks occupying rice culture habitats. Wilson Bull. 93:551-554.
20. Brackney, A. W., and J. W. Hupp. 1993. Autumn diet of lesser snow geese staging in northeastern Alaska. J. Wildl. Manag. 57:55-61.
21. Brackney, A. W., and R. M. Platte. 1987. Habitat use and behavior of molting Oldsquaw on the coast of the Arctic National Wildlife Refuge. ANWR Prog. Rep. No. FY86-17. Arctic National Wildlife Refuge, USFWS, Fairbanks, AK.
22. Brand, D. J. 1961. A Comparative Study of the Cape Teal (*Anas Capensis* Gmelin) and the Cape Shoveller (*Spatula Capensis* Eyton): With Special Reference to Breeding Biology, Development and Food Requirements. Ph.D. Thesis. University of South Africa.
23. Brickhill, J. 1987. The conservation status of Malleefowl in New South Wales. Master’s Thesis. Univ. New England, New England, Armidale, NSW. RAOU Microfiche Ser. 36.
24. Briggs, S. V. 1982. Food habits of the Freckled Duck and associated waterfowl in north-western New South Wales. Wildfowl. 33:88-93.
25. Briggs, S. V., M. T. Maher, and R. P. Palmer. 1985. Bias in food habits of Australian waterfowl. Wildl. Res. 12:507-514.
26. Brown, P. W., and L. H. Fredrickson. 1986. Food habits of breeding white-winged scoters. Can. J. Zool. 64:1652-1654.
27. Bruzual, J., and I. Bruzual. 1983. Feeding habits of whistling ducks in the Calabozo Ricefields, Venezuela, during the non-reproductive period. Wildfowl. 34:20-26.
28. Budeau, D. A., J. T. Ratti, and C. R. Ely. 1991. Energy dynamics, foraging ecology, and behavior of prenesting greater white-fronted geese. J. Wildl. Manag. 55:556-563.
29. Bur, M. T., M. A. Stapanian, G. Bernhardt, and M. W. Turner. 2008. Fall diets of red-breasted merganser (*Mergus serrator*) and walleye (*Sander vitreus*) in Sandusky Bay and adjacent waters of western Lake Erie. Am. Midl. Nat. 159:147-161.
30. Bustnes, J. O., M. Asheim, T. H. Bjørn, H. Gabrielsen, and G. H. Systad. 2000. The diet of Steller’s Eiders wintering in Varangerfjord, northern Norway. Wilson Bull. 112:8-13.
31. Buxton, N. E., and C. M. Young. 1981. The food of the Shelduck in north-east Scotland. Bird Study. 28:41-48.
32. Bédard, J., and G. Gauthier. 1989. Comparative energy budgets of Greater Snow Geese *Chen caerulescens atlantica* staging in two habitats in spring. Ardea. 77:3-20.
33. Cadieux, M., G. Gauthier, R. J. Hughes, and A. E. Burger. 2005. Feeding ecology of Canada Geese (*Branta canadensis interior*) in sub-arctic inland tundra during brood-rearing. Auk. 122:144-157.
34. Cantin, M., J. Bedard, and H. Milne. 1974. The food and feeding of common eiders in the St. Lawrence estuary in summer. Can. J. Zool. 52:319-334.
35. Caziani, S. M., and J. J. Protomastro. 1994. Diet of the chaco chachalaca. Wilson Bull. 106:640-648.
36. Collier, K. J. 1991. Invertebrate food supplies and diet of blue duck on rivers in two regions of the North Island, New Zealand. N. Z. J. Ecol. 15:131-138.
37. Corti, P., and R. P. Schlatter. 2002. Feeding ecology of the black-necked swan *Cygnus melancoryphus* in two wetlands of Southern Chile. Stud. Neotrop. Fauna Environ. 37:9-14.
38. Costanzo, G. R. 1988. Wintering ecology of black ducks along coastal New Jersey. Ph.D. Thesis. Cornell Univ., Ithaca, NY.
39. Cottam, C. 1939. Food habits of North American diving ducks. United States Department of Agriculture, Economic Research Service.
40. Cottam, C., and F. M. Uhler. 1945. Birds in relation to fishes. US Department of the Interior, Fish and Wildlife Service.
41. Coulter, M. W. 1955. Spring food habits of surface-feeding ducks in Maine. J. Wildl. Manag. 19:263-267.
42. Craven, S. R., and R. A. Hunt. 1984. Fall food habits of Canada geese in Wisconsin. J. Wildl. Manag. 48:169-173.
43. Cronan, J. M, Jr. 1957. Food and feeding habits of the scaups in Connecticut waters. Auk. 74:459-468.
44. Delroy, L. B. 1974. The food of waterfowl (Anatidae) in the Southern Coorong saltwater habitat of south Australia. S. Aust. Ornithol. 27:157-163.
45. Dementiev, G. P., and N. A. Gladkov. 1952. Birds of the Soviet Union. Israel Program for Scientific Translations. US Dept Interior and National Science Foundation, Washington.
46. Douthwaite, R. J. 1977. Filter-feeding ducks of the Kafue Flats, Zambia, 1971-1973. Ibis. 119:44-66.
47. Douthwaite, R. J. 1978. Geese and Red-knobbed Coot on the Kafue Flats in Zambia, 1970-1974. Afr. J. Ecol. 16:29-47.
48. Drobney, R. D., and L. H. Fredrickson. 1979. Food selection by wood ducks in relation to breeding status. J. Wildl. Manag. 43:109-120.
49. DuBowy, P. J. 1985. Feeding ecology and behavior of postbreeding male blue-winged teal and northern shovelers. Can. J. Zool. 63:1292-1297.
50. Earnst, S. L. 1992. Behavior and ecology of Tundra Swans during summer, autumn, and winter. Ph.D. Thesis. Ohio State University.
51. Eccleston, K. A. 1999. Food sources as a factor in the decline of greater scaup and lesser scaup ducks. Master’s Thesis. University of Connecticut.
52. Eisenhauer, D. I., and C. M. Kirkpatrick. 1977. Ecology of the emperor goose in Alaska. Wildl. Monogr.:3-62.
53. Ely, C. R., and D. G. Raveling. 2011. Seasonal variation in nutritional characteristics of the diet of greater white-fronted geese. J. Wildl. Manag. 75:78-91.
54. Erard, C., M. Théry, and D. Sabatier. 1991. Régime alimentaire de *Tinamus major* (Tinamidae), *Crax alector* (Cracidae) et *Psophia crepitans* (Psophiidae), en forêt guyanaise. Gib. Faun. Sauv. 8:183-210.
55. Erskine, A. J. 1972. Buffleheads. Can. Wildl. Serv., Ottawa. Monogr. Ser. No. 4.
56. Euliss, N. H. Euliss, N. H, Jr., and S. W. Harris. 1987. Feeding ecology of northern pintails and green-winged teal wintering in California. J. Wildl. Manag. 51:724-732.
57. Euliss, N. H., R. L. Jarvis, and D. S. Gilmer. 1997. Relationship between waterfowl nutrition and condition on agricultural drainwater ponds in the Tulare Basin, California: waterfowl body composition. Wetlands. 17:106-115.
58. Evans-Peters, S. T. 2013. The ecology of wild turkey (*Meleagris gallopavo*) foraging in Pacific Northwest ecosystems. Ph.D. Thesis. Oregon State University.
59. Feltham, M. J. 1990. The diet of red-breasted mergansers (*Mergus serrator*) during the smolt run in NE Scotland: the importance of salmon (*Salmo salar*) smolts and parr. J. Zool. (Lond.). 222:285-292.
60. Fischer, J. B., and C. R. Griffin. 2000. Feeding behavior and food habits of wintering Harlequin Ducks at Shemya Island, Alaska. Wilson Bull. 112:318-325.
61. Fitzner, R. E., and R. H. Gray. 1994. Winter diet and weights of Barrow’s and common goldeneye in southcentral Washington. Northwest. Sci. 68:172-177.
62. Fox, A. D. 1993. Pre-nesting feeding selectivity of Pink-footed Geese *Anser brachyrhynchus* in artificial grasslands. Ibis. 135:417-423.
63. Fox, A. D., J. Kahlert, and H. Ettrup. 1998. Diet and habitat use of moulting Greylag Geese *Anser anser* on the Danish island of Saltholm. Ibis. 140:676-683.
64. Fox, A. D., and E. Bergersen. 2005. Lack of competition between barnacle geese *Branta leucopsis* and pink-footed geese *Anser brachyrhynchus* during the pre-breeding period in Svalbard. J. Avian Biol. 36:173-178.
65. Fried, L. A. 1940. The Food Habits of the Ring-Necked Pheasant in Minnesota. J. Wildl. Manag. 4:27-36.
66. Frith, H. J. 1959. The ecology of wild ducks in inland New South Wales. III. Food habits. Wildl. Res. 4:131-155.
67. Frith, H. J. 1982. Waterfowl in Australia. East-West Center Press Honolulu.
68. Frith, H. J., L. W. Braithwaite, and J. L. McKean. 1969. Waterfowl in an inland swamp in New South Wales. 2. Food. Wildl. Res. 14:17-64.
69. Frith, H. J., and S. Davies. 1961. Ecology of the Magpie Goose, *Anseranas semipalmata* Latham (Anatidae). Wildl. Res. 6:91-141.
70. Fuentes, C., M. I. Sánchez, N. Selva, and A. J. Green. 2004. The diet of the marbled Teal *Marmoreneta angustirostris* in southern Alicante, Eastern spain. Rev. Écol. 59:475-490.
71. Gaines, W. L., and R. E. Fitzner. 1987. Winter diet of the harlequin duck at Sequim Bay, Puget Sound, Washington. Northwest. Sci. 61:213-215.
72. Gammonley, J. H. 1995. Spring feeding ecology of Cinnamon Teal in Arizona. Wilson Bull. 107:64-72.
73. Gammonley, J. H., and M. E. Heitmeyer. 1990. Behavior, body condition, and foods of buffleheads and lesser scaups during spring migration through the Klamath Basin, California. Wilson Bull. 102:672-683.
74. Gammonley, J. H., and M. K. Laubhan. 2002. Patterns of food abundance for breeding waterbirds in the San Luis Valley of Colorado. Wetlands. 22:499-508.
75. Gates, R. J., D. F. Caithamer, W. E. Moritz, and T. C. Tacha. 2001. Bioenergetics and nutrition of Mississippi Valley population Canada geese during winter and migration. Wildl. Monogr. 146:1-65.
76. Gauthier, G. 1993. Feeding ecology of nesting Greater Snow Geese. J. Wildl. Manag. 57:216-223.
77. Gill, J. A., A. R. Watkinson, and W. J. Sutherland. 1997. Causes of the redistribution of Pink-footed Geese *Anser brachyrhynchus* in Britain. Ibis. 139:497-503.
78. Giroux, J., and J. Bédard. 1988. Age differences in the fall diet of greater snow geese in Quebec. Condor. 90:731-734.
79. Giroux, J., and R. Bergeron. 1996. Spring diets of sympatric greater snow geese and Canada geese in southern Quebec. Can. J. Zool. 74:950-953.
80. Glading, B., H. H. Biswell, and C. F. Smith. 1940. Studies on the food of the California quail in 1937. J. Wildl. Manag. 4:128-144.
81. Gloutney, M. L., R. T. Alisauskas, A. D. Afton, and S. M. Slattery. 2001. Foraging time and dietary intake by breeding Ross’s and Lesser Snow Geese. Oecologia (Berl.). 127:78-86.
82. Glover, F. A., and R. W. Bailey. 1949. Wild turkey foods in West Virginia. J. Wildl. Manag. 13:255-265.
83. González-Acuña, D., P. Riquelme-Salasar, J. Cruzatt-Molina, P. López-Sepúlveda, L. Moreno-Salas, and R. Figueroa-Rojas. 2013. Dieta de la codorniz californiana (*Callipepla californica*) en áreas agrícolas del centro sur de Chile. Rev. Cient. 23:312-317.
84. Goudie, R. I., and C. D. Ankney. 1986. Body size, activity budgets, and diets of sea ducks wintering in Newfoundland. Ecology (N. Y.). 67:1475-1482.
85. Gowland, P. N. 1988. RAOU Microfiche 35.
86. Green, A. J., and M. I. Sánchez. 2003. Spatial and temporal variation in the diet of Marbled Teal *Marmaronetta angustirostris* in the western Mediterranean: This globally threatened species is less dependent on invertebrates and more dependent on seeds than other ducks. Bird Study. 50:153-160.
87. Halse, S. A. 1984. Diet, body condition, and gut size of Egyptian geese. J. Wildl. Manag. 48:569-573.
88. Havera, S. P. 1998. Waterfowl of Illinois: status and management. Phoenix Publishing Urbana, IL.
89. Hayes, F., C. Shameerudeen, B. Sanasie, B. D. Hayes, C. Ramjohn, and F. Lucas. 2009. Ecology and behaviour of the critically endangered Trinidad piping-guan *Aburria pipile*. Endang. Species Res. 6:223-229.
90. Hockey, P., W. R. J. Dean, and P. Ryan, eds. 2005. Roberts birds of southern Africa. Trustees of the John Voelcker Bird Book Fund.
91. Hohman, W. L. 1985. Feeding ecology of ring-necked ducks in northwestern Minnesota. J. Wildl. Manag. 49:546-557.
92. Hohman, W. L., D. W. Woolington, and J. H. Devries. 1990. Food habits of wintering canvasbacks in Louisiana. Can. J. Zool. 68:2605-2609.
93. Hohman, W. L., T. M. Stark, and J. L. Moore. 1996. Food availability and feeding preferences of breeding fulvous whistling-ducks in Louisiana ricefields. Wilson Bull. 108:137-150.
94. Hohman, W. L., and C. D. Ankney. 1994. Body size and condition, age, plumage quality, and foods of prenesting male cinnamon teal in relation to pair status. Can. J. Zool. 72:2172-2176.
95. Hoppe, R. T., L. M. Smith, and D. B. Wester. 1986. Foods of wintering diving ducks in South Carolina. J. Field Ornithol. 57:126-134.
96. Hughes, J. H., and E. L. Young. 1982. Autumn foods of dabbling ducks in southeastern Alaska. J. Wildl. Manag. 46:259-263.
97. James, H. F., and D. A. Burney. 1997. The diet and ecology of Hawaii’s extinct flightless waterfowl: evidence from coprolites. Biol. J. Linn. Soc. 62:279-297.
98. Jamieson, S. E., G. J. Robertson, and H. G. Gilchrist. 2001. Autumn and winter diet of long-tailed duck in the Belcher Islands, Nunavut, Canada. Waterbirds. 24:129-132.
99. Jarvis, R. L., and J. H. Noyes. 1986. Foods of canvasbacks and redheads in Nevada: paired males and ducklings. J. Wildl. Manag. 50:199-203.
100. Johnsgard, P. A., ed. 1978. Ducks, geese, and swans of the world. University of Nebraska Press Lincoln, NE.
101. Johnson, S. R. 1984. Prey selection by Oldsquaws in a Beaufort Sea lagoon, Alaska. Pp. 12-19 *in* Sanger, G. A., and P. F. Springer, eds. Marine birds: their feeding ecology and commercial fisheries relationships. Can. Wildl. Serv. Spec. Publ., Ottawa, ON.
102. Jones, J. J., and R. D. Drobney. 1986. Winter feeding ecology of scaup and common goldeneye in Michigan. J. Wildl. Manag. 50:446-452.
103. Jonkel, C. J., and K. R. Greer. 1963. Fall food habits of spruce grouse in northwest Montana. J. Wildl. Manag. 27:593-596.
104. Jorde, D. G., and R. B. Owen, Jr. 1990. Food of black ducks, Anas rubripes, wintering in marine habitats of Maine. Can. Field-Nat. 104:300-302.
105. Karmiris, I., S. Kazantzidis, and M. Panagiotopoulou. 2009. A note on the diet of the Lesser White-fronted Goose wintering in the Evros Delta, Greece. Pp. 68-70 *in* Conservation of Lesser White-fronted Goose on the European migration route — Final report of the EU LIFE-Nature project 2005–2009.
106. Kear, J., ed. 2005. Bird families of the world: ducks, geese and swans. Oxford University Press Oxford, UK.
107. Kenow, K. P., and D. H. Rusch. 1996. Food Habits of Redheads at the Horicon Marsh, Wisconsin. J. Field Ornithol. 67:649-659.
108. Kentish, B., and M. Westbrooke. 1993. Crop and gizzard contents of a road-dead Malleefowl. Emu. 94:130-132.
109. Kingsford, R. T. 1989. Food of the Maned Duck *Chenonetta jubata* during the breeding season. Emu. 89:119-124.
110. Koehl, P. S., T. C. Rothe, and D. V. Derksen. 1982. Winter food habits of Barrow’s Goldeneyes in southeast Alaska. Pp. 12-19 *in* Nettleship, D. N., G. A. Sanger, and P. F. Springer, eds. Marine birds: their feeding ecology and commercial fisheries relationships. Can. Wildl. Serv. Spec. Publ., Ottawa, ON.
111. Kopischke, E. D., and S. W. Harris. 1969. Food habits of Minnesota pheasants. Loon. 41:119-123.
112. Korschgen, C. E., L. S. George, and W. L. Green. 1988. Feeding ecology of canvasbacks staging on Pool 7 of the Upper Mississippi River. Pp. 237-249 *in* Weller, M. W., ed. Waterfowl in winter. University of Minnesota Press, Minneapolis, MN.
113. Kramer, G. W., and N. H. Euliss, Jr. 1986. Winter foods of black-bellied whistling-ducks in northwestern Mexico. J. Wildl. Manag. 50:413-416.
114. Krapu, G. L., K. J. Reinecke, D. G. Jorde, and S. G. Simpson. 1995. Spring-staging ecology of midcontinent greater white-fronted geese. J. Wildl. Manag. 59:736-746.
115. Kumssa, T., and A. Bekele. 2013. Population status, feeding ecology and activity pattern of helmeted guinea fowl (*Numidia* [sic] *meleagris*) in Abijata-Shalla Lakes National Park. Afr. J. Environ. Sci. Technol. 7:49-55.
116. Landers, J. L., A. S. Johnson, P. H. Morgan, and W. P. Baldwin. 1976. Duck foods in managed tidal impoundments in South Carolina. J. Wildl. Manag. 40:721-728.
117. Landers, J. L., T. T. Fendley, and A. S. Johnson. 1977. Feeding ecology of wood ducks in South Carolina. J. Wildl. Manag. 41:118-127.
118. Lavery, H. J. 1971. Studies of waterfowl (Anatidae) in north Queensland. 6. Feeding methods and foods. Qld. J. Agric. Anim. Sci. 28:255-273.
119. Leonard, J. W., and D. S. Shetter. 1937. Studies on merganser depredations in Michigan trout waters. Trans. Am. Fish. Soc. 66:335-337.
120. Londoño, G. A., M. C. Muñoz, and M. M. Rios. 2007. Density and natural history of the Sickle-winged Guan (*Chamaepetes goudotii*) in the Central Andes, Colombia. Wilson J. Ornithol. 119:228-238.
121. Lovvorn, J. R., S. E. Richman, J. M. Grebmeier, and L. W. Cooper. 2003. Diet and body condition of spectacled eiders wintering in pack ice of the Bering Sea. Polar Biol. 26:259-267.
122. Mabbott, D. C. 1920. Food habits of seven species of American shoal-water ducks. U S Dep. Agric. Agric. Bull. 862.
123. Madriz, M. 1983. Food habits of the Brazilian duck in Apure State, Venezuela. J. Wildl. Manag. 47:531-533.
124. Marchant, S., and P. J. Higgins, eds. 1990. Handbook of Australian, New Zealand and Antarctic birds. Oxford University Press Oxford, UK.
125. Marion, W. R. 1976. Plain Chachalaca food habits in south Texas. Auk. 93:376-379.
126. Markkola, J., N. Marika, and S. Rytkönen. 2003. Diet selection of lesser white-fronted geese *Anser erythropus* at a spring staging area. Ecography. 26:705-714.
127. Marriott, R. W., and D. K. Forbes. 1970. The digestion of lucerne chaff by Cape Barren geese, *Cereopsis novaehollandiae* Latham. Aust. J. Zool. 18:257-263.
128. McCaw III, J. H., P. J. Zwank, and R. L. Steiner. 1996. Abundance, Distribution, and Behavior of Common Mergansers Wintering on a Reservoir in Southern New Mexico. J. Field Ornithol. 67:669-679.
129. McGilvrey, F. B. 1967. Food habits of sea ducks from the north-eastern United States. Wildfowl. 18:142-145.
130. McKelvey, R. W. 1981. Some aspects of the winter feeding ecology of trumpeter swans at Port Alberni and Comox Harbour, British Columbia. Ph.D. Thesis. Simon Fraser University.
131. McMahan, C. A. 1970. Food habits of ducks wintering on Laguna Madre, Texas. J. Wildl. Manag. 34:946-949.
132. Meininger, P. L., and H. Snoek. 1992. Non-breeding Shelduck Tadorna tadorna in the southwest Netherlands: effects of habitat changes on distribution, numbers, moulting sites and food. Wildfowl. 43:139-151.
133. Merkel, F. R., S. E. Jamieson, K. Falk, and A. Mosbech. 2007. The diet of common eiders wintering in Nuuk, Southwest Greenland. Polar Biol. 30:227-234.
134. Metzner, K. A. 1993. Ecological strategies of wintering Steller’s eiders on Izembek Lagoon and Cold Bay, Alaska. Master’s Thesis. University of Missouri-Columbia.
135. Middleton, B. A., and A. G. van der Valk. 1987. The food habits of greylag and barheaded geese in the Keoladeo National Park, India. Wildfowl. 38:94-102.
136. Migoya, R., and G. A. Baldassarre. 1993. Harvest and food habits of waterfowl wintering in Sinaloa, Mexico. Southwest. Nat. 38:168-171.
137. Miller, M. R. 1987. Fall and winter foods of northern pintails in the Sacramento Valley, California. J. Wildl. Manag. 51:405-414.
138. Miller, S. W., and J. S. Barclay. 1973. Predation in warm water reservoirs by wintering common mergansers. Pp. 243-252 *in* Proceedings of the 27th annual conference of the Southeastern Association of Game and Fish Commissioners. Vol. 27.
139. Mitchell, R. 1983. Preliminary feeding data on red-billed teal, Cape teal and Cape shoveller at Barberspan. S. Afr. J. Wildl. Res. 13:47-48.
140. Moore, S. J., P. F. Battley, I. M. Henderson, and C. J. Webb. 2006. The diet of brown teal (*Anas chlorotis*). N. Z. J. Ecol. 30:397.
141. Munro, J. 1939. A. 1939. Studies of waterfowl in British Columbia: Barrow’s goldeneye, American goldeneye. Trans. Royal Can. Inst. 24:259-318.
142. Munro, J. A., and W. A. Clemens. 1932. Food of the American Merganser (*Mergus merganser americanus*) in British Columbia: A preliminary paper. Can. Field-Nat. 46:166-168.
143. Muñoz, M. C., and G. H. Kattan. 2007. Diets of cracids: how much do we know. Ornitol. Neotrop. 18:21-36.
144. Naranjo, L. G. 1986. Aspects of the biology of the horned screamer in southwestern Colombia. Wilson Bull. 98:243-256.
145. Nechaev, V. A. 1992. Status of the Swan Goose and the Mandarin Duck on Sakhalin Island, Russian Far East. IWRB Threat. Waterfowl Res. Group Newsl. 2:12-14.
146. Njiforti, H. L., L. Hebou, and A. Bodenkamp. 1998. Diet of the helmeted guineafowl (*Numida meleagris galeata* Pallas) in the Waza region of North Cameroon. Afr. J. Ecol. 36:71-82.
147. Norman, F. I., and L. Mumford. 1982. Food of the Chestnut Teal, Anas Castanea, in the Gippsland Lakes Region of Victoria. Wildl. Res. 9:151-155.
148. Noyes, J. H., and R. L. Jarvis. 1985. Diet and nutrition of breeding female redhead and canvasback ducks in Nevada. J. Wildl. Manag. 49:203-211.
149. Olney, P. 1963. The food and feeding habits of Tufted Duck *Aythya fuligula*. Ibis. 105:55-62.
150. Olney, P. 1965. The food and feeding habits of Shelduck *Tadorna tadorna*. Ibis. 107:527-532.
151. Olney, P. 1968. The food and feeding-habits of the Pochard, *Aythya ferina*. Biol. Conserv. 1:71-76.
152. Olney, P., and D. H. Mills. 1963. The food and feeding habits of goldeneye *Bucephala clangula* in Great Britain. Ibis. 105:293-300.
153. Owen, M. 1973. The winter feeding ecology of Wigeon at Bridgwater Bay, Somerset. Ibis. 115:227-243.
154. Owen, M. 1975. An assessment of fecal analysis technique in waterfowl feeding studies. J. Wildl. Manag. 39:271-279.
155. Owen, M. 1976. The Selection of Winter Food by Whiterfronted Geese. J. Appl. Ecol. 13:715-729.
156. Owen, M., and C. J. Cadbury. 1975. The ecology and mortality of swans at the Ouse Washes, England. Wildfowl. 26:31-42.
157. Owen, M., and G. J. Thomas. 1979. The feeding ecology and conservation of wigeon wintering at the Ouse Washes, England. J. Appl. Ecol. 16:795-809.
158. Owen, M., and R. H. Kerbes. 1971. On the autumn food of barnacle geese at Caerlaverock National Nature Reserve. Wildfowl. 22:114-119.
159. Pacheco, S. 1994. Hábitos alimentarios y uso estacional de hábitat de la pava crestada (*Penelope purpurascens*) en el bosque seco tropical, Parque Nacional Santa Rosa, Costa Rica. Master’s Thesis. Universidad Nacional, Heredia, Costa Rica.
160. Palmer, R. S., ed. 1976. Handbook of North American birds. Vols. 2,3 Yale University Press New Haven, CT.
161. Paulus, S. L. 1982. Feeding ecology of gadwalls in Louisiana in winter. J. Wildl. Manag. 46:71-79.
162. Pendergast, B. A., and D. A. Boag. 1970. Seasonal changes in diet of spruce grouse in central Alberta. J. Wildl. Manag. 34:605-611.
163. Perry, M. C., P. C. Osenton, and E. Lohnes. 2004. Food habits of mute swans in Chesapeake Bay. Pp. 31-36 *in* Perry, M. C., ed. Mute Swans and their Chesapeake Bay Habitats: Proceedings of a Symposium: US Geological Survey, Biological Resources Discipline Information and Technology Report USGS/BRD/ITR-2004-0005.
164. Perry, M. C., and F. M. Uhler. 1982. Food habits of diving ducks in the Carolinas. Pp. 492-504 *in* Proc. Southeast. Assoc. Fish Wildl. Agencies. Vol. 36.
165. Perry, M. C., and F. M. Uhler. 1988. Food habits and distribution of wintering canvasbacks, *Aythya valisineria*, on Chesapeake Bay. Estuaries. 11:57-67.
166. Peters, M. S., and A. D. Afton. 1993. Diets of ring-necked ducks wintering on Catahoula Lake, Louisiana. Southwest. Nat. 38:166-168.
167. Petersen, M. R., J. F. Piatt, and K. A. Trust. 1998. Foods of spectacled eiders Somateria fischeri in the Bering Sea, Alaska. Wildfowl. 49:124-128.
168. Peterson, S. R., and R. S. Ellarson. 1977. Food habits of Oldsquaws wintering on Lake Michigan. Wilson Bull. 89:81-91.
169. Petrie, S. A. 1996. Red-billed teal foods in semiarid South Africa: a north-temperate contrast. J. Wildl. Manag. 60:874-881.
170. Petrie, S. A. 2000. Winter and spring foods of white-faced whistling ducks in northern KwaZulu-Natal, South Africa. S. Afr. J. Wildl. Res. 30.
171. Poole, A., ed. 2005. The Birds of North America Online. Cornell Laboratory of Ornithology Ithaca, NY URL http://bna.birds.cornell.edu/BNA/.
172. Prevett, J. P., I. F. Marshall, and V. G. Thomas. 1979. Fall foods of Lesser Snow Geese in the James Bay region. J. Wildl. Manag. 43:736-742.
173. Prevett, J. P., I. F. Marshall, and V. G. Thomas. 1985. Spring foods of Snow and Canada Geese at James Bay. J. Wildl. Manag. 49:558-563.
174. Prop, J., M. R. van Eerden, and R. H. Drent. 1984. Reproductive success of the barnacle goose *Branta leucopsis* in relation to food exploitation on the breeding grounds, western Spitsbergen. Nor. Polarinst. Skr. 181:87-117.
175. Prop, J., and C. Deerenberg. 1991. Spring staging in brent geese *Branta bernicla*: feeding constraints and the impact of diet on the accumulation of body reserves. Oecologia (Berl.). 87:19-28.
176. Prop, J., and J. de Vries. 1993. Impact of snow and food conditions on the reproductive performance of barnacle geese *Branta leucopsis*. Ornis Scand. 24:110-121.
177. Prop, J., and J. M. Black. 1998. Food intake, body reserves and reproductive success of barnacle geese *Branta leucopsis* staging in different habitats. Nor. Polarinst. Skr. 200:175-194.
178. Ranwell, D. S., and B. M. Downing. 1959. Brent goose *(Branta bernicla* L.) winter feeding pattern and zostera resources at Scolt Head Island, Norfolk. Anim. Behav. 7:42-56.
179. Rawls, C. K, Jr. 1954. Reelfoot Lake waterfowl research. Unpubl. Rep.
180. Reed, A., R. Benoit, M. Julien, and R. Lalumiere. 1996. Goose use of the coastal habitats of northeastern James Bay. Can. Wildl. Ser. Occas. Pap. 92.
181. Reinecke, K. J., and R. B. Owen, Jr. 1980. Food use and nutrition of black ducks nesting in Maine. J. Wildl. Manag. 44:549-558.
182. Rofritz, D. J. 1977. Oligochaeta as a winter food source for the Old Squaw. J. Wildl. Manag. 41:590-591.
183. Ross, R. K., S. A. Petrie, S. S. Badzinski, and A. Mullie. 2005. Autumn diet of greater scaup, lesser scaup, and long-tailed ducks on eastern Lake Ontario prior to zebra mussel invasion. Wildl. Soc. Bull. 33:81-91.
184. Salyer, J. C., and K. F. Lagler. 1940. The food and habits of the American merganser during winter in Michigan, considered in relation to fish management. J. Wildl. Manag. 4:186-219.
185. Santamaría, M., and A. M. Franco. 2000. Frugivory of Salvin’s Curassow in a rainforest of the Colombian Amazon. Wilson Bull. 112:473-481.
186. Saunders, G. B., and D. C. Saunders. 1981. Waterfowl and their wintering grounds in Mexico, 1937-1964. U S Fish Wildl. Serv. Resour. Publ. 138.
187. Schemnitz, S. D. 1956. Wild turkey food habits in Florida. J. Wildl. Manag. 20:132-137.
188. Schmutz, J. A. 1994. Age, habitat and tide effects on feeding activity of Emperor Geese during autumn migration. Condor. 96:46-51.
189. Sell, D. L. 1979. Fall foods of teal on the Texas High Plains. Southwest. Nat. 24:373-375.
190. Serie, J. R., and G. A. Swanson. 1976. Feeding ecology of breeding gadwalls on saline wetlands. J. Wildl. Manag. 40:69-81.
191. Sherwood, G. A. 1960. The Whistling Swan in the west with particular reference to Great Salt Lake Valley, Utah. Condor. 62:370-377.
192. Squires, J. R., and S. H. Anderson. 1995. Trumpeter swan (*Cygnus buccinator*) food habits in the Greater Yellowstone Ecosystem. Am. Midl. Nat. 133:274-282.
193. Stewart, R. E., and J. H. Manning. 1958. Distribution and ecology of Whistling Swans in the Chesapeake Bay region. Auk. 75:203-212.
194. Stott, R. S., and D. P. Olson. 1973. Food-habitat relationship of sea ducks on the New Hampshire coastline. Ecology (N. Y.). 54:996-1007.
195. Stoudt, J. H. 1944. Food preferences of mallards on the Chippewa National Forest, Minnesota. J. Wildl. Manag. 8:100-112.
196. Stromborg, K. L. 1979. Pheasant food habits in spring and consumption of seed treatment pesticides. J. Wildl. Manag. 43:214-219.
197. Sugden, L. G., and E. A. Driver. 1980. Natural foods of mallards in Saskatchewan parklands during late summer and fall. J. Wildl. Manag. 44:705-709.
198. Summers, R. W., and A. Grieve. 1982. Diet, feeding behaviour and food intake of the upland goose (*Chloephaga picta*) and ruddy-headed goose (*C. rubidiceps*) in the Falkland Islands. J. Appl. Ecol. 19:783-804.
199. Swanson, G. A., G. L. Krapu, and J. R. Serie. 1979. Foods of laying female dabbling ducks on the breeding grounds. Pp. 47-57 *in* Bookhout, T. A., ed. Waterfowl and wetlands-an integrated review. Wildlife Society, Madison, WI.
200. Swanson, G. A., M. I. Meyer, and J. R. Serie. 1974. Feeding ecology of breeding blue-winged teals. J. Wildl. Manag. 38:396-407.
201. Swanson, G. A., M. I. Meyer, and V. A. Adomaitis. 1985. Foods consumed by breeding mallards on wetlands of south-central North Dakota. J. Wildl. Manag. 49:197-203.
202. Swanson, G. A., and M. I. Meyer. 1977. Impact of fluctuating water levels on feeding ecology of breeding blue-winged teal. J. Wildl. Manag. 41:426-433.
203. Swiderek, P. K., A. S. Johnson, P. E. Hale, and R. L. Joyner. 1988. Production, management, and waterfowl use of sea purslane, Gulf Coast muskgrass, and widgeongrass in brackish impoundments. Pp. 441-457 *in* Weller, M. W., ed. Waterfowl in winter. University of Minnesota Press, Minneapolis, MN.
204. Talukdar, B. K. 2003. Food and Feeding Habitat of the White-winged Duck in Assam. Pp. 76-77 *in* Hughes, B., ed. Threatened Waterfowl Specialist Group News. No. 14.
205. Taylor, T. S. 1978. Spring foods of migrating blue-winged teals on seasonally flooded impoundments. J. Wildl. Manag. 42:900-903.
206. Thomas, V. G. 1984. Winter diet and intestinal proportions of rock and willow ptarmigan and sharp-tailed grouse in Ontario. Can. J. Zool. 62:2258-2263.
207. Thompson, D. 1973. Feeding ecology of diving ducks on Keokuk Pool, Mississippi River. J. Wildl. Manag. 37:367-381.
208. Thompson, J. D., B. J. Sheffer, and G. A. Baldassarre. 1992. Food habits of selected dabbling ducks wintering in Yucatán, Mexico. J. Wildl. Manag. 56:740-744.
209. Thompson, J. E. 1996. Comparative reproductive ecology of female buffleheads (*Bucephala albeola*) and Barrow’s goldeneyes (*Bucephala islandica*) in central British Columbia. Ph.D. Thesis. Univ. of Western Ontario, London, ON.
210. Thompson, J. E., and R. D. Drobney. 1997. Diet and nutrition of male canvasbacks during postreproductive molts. J. Wildl. Manag. 61:426-434.
211. Tietje, W. D., and J. G. Teer. 1996. Winter feeding ecology of northern shovelers on freshwater and saline wetlands in south Texas. J. Wildl. Manag. 60:843-855.
212. Turnbull, R. E., F. A. Johnson, and D. H. Brakhage. 1989. Status, distribution, and foods of fulvous whistling-ducks in south Florida. J. Wildl. Manag. 53:1046-1051.
213. Veltman, C. J., K. J. Collier, I. M. Henderson, and L. Newton. 1995. Foraging ecology of blue ducks *Hymenolaimus malacorhynchos* on a New Zealand river: Implications for conservation. Biol. Conserv. 74:187-194.
214. Vermeer, K. 1983. Diet of the Harlequin Duck in the Strait of Georgia, British Columbia. Murrelet. 64:54-57.
215. Vermeer, K., and C. D. Levings. 1977. Populations, biomass and food habits of ducks on the Fraser Delta intertidal area, British Columbia. Wildfowl. 28:49-60.
216. Vermeer, K., and N. Bourne. 1984. The White-winged Scoter diet in British Columbia waters: resource partitioning with other scoters. Pp. 30-38 *in* Nettleship, D. N., G. A. Sanger, and P. F. Springer, eds. Marine birds: their feeding ecology and commercial fisheries relationships. Can. Wildl. Serv. Spec. Publ., Ottawa, ON.
217. Watson, R. M. 1970. The diet of duck and coot on Lake Naivasha. Afr. J. Ecol. 8:131-144.
218. Weeden, R. B. 1969. Foods of rock and willow ptarmigan in central Alaska with comments on interspecific competition. Auk. 86:271-281.
219. Weller, M. W. 1972. Ecological studies of Falkland Islands’ waterfowl. Wildfowl. 23:25-44.
220. Weller, M. W. 1975. Ecology and behaviour of the South Georgia Pintail *Anas g. georgica*. Ibis. 117:217-231.
221. White, H. C. 1957. Food and natural history of Mergansers on salmon waters in the maritime provinces of Canada. Fisheries Research Board of Canada Ottawa, ON.
222. Winterbottom, J. M. 1974. The Cape Teal. Ostrich. 45:110-132.
223. Wishart, R. A. 1983. The behavioral ecology of the American Wigeon (*Anas americana*) over its annual cycle. Ph.D. Thesis. Univ. of Manitoba, Winnipeg, MB.
224. Woodin, M. C., and G. A. Swanson. 1989. Foods and dietary strategies of prairie-nesting Ruddy Ducks and Redheads. Condor. 91:280-287.
225. Woodyard, E. R., and E. G. Bolen. 1984. Ecological studies of muscovy ducks in Mexico. Southwest. Nat. 29:453-461.
226. Yocum, C. F., and M. Keller. 1961. Correlation of food habits and abundance of waterfowl, Humboldt Bay, California. Calif. Fish Game. 47:41-54.
227. Zhang, J., and J. Lu. 1999. Feeding ecology of two wintering geese species at Poyang Lake, China. J. Freshw. Ecol. 14:439-445.
